# Supplementary material for: Carbohydrate-aromatic interface and molecular architecture of lignocellulose
Source: Nat Commun. 2022 Jan 27;13:538. doi: 10.1038/s41467-022-28165-3 (PMC8795156; doi:10.1038/s41467-022-28165-3)
Supplement: Supplementary file 1 — Supplementary Information [file 41467_2022_28165_MOESM1_ESM.pdf]

Supplementary Information

Carbohydrate-Aromatic Interface and Molecular Architecture of Lignocellulose

Alex Kirui[1#], Wancheng Zhao[1#], Fabien Deligey[1#], Hui Yang[2], Xue Kang[1], Frederic Mentink-Vigier[3], Tuo Wang[1]

[1] Department of Chemistry, Louisiana State University, Baton Rouge, LA 70803, USA

[2] Department of Biology, Pennsylvania State University, University Park, PA 16802, USA

[3] National High Magnetic Field Laboratory, Tallahassee, FL 32310, USA

# These authors contributed equally

Table of Contents

Supplementary Figure 1. 2D ^{13}C-^{13}C spectra of polysaccharides in softwood and hardwood 3
Supplementary Figure 2. Cellulose bundling models that violate the NMR constraints 4
Supplementary Figure 3: Lignin distributed in the mobile and rigid phases 5
Supplementary Figure 4. Solution NMR of lignin and polysaccharides in woody stems 6
Supplementary Figure 5. Spectral deconvolution reveals the lignin composition 7
Supplementary Figure 6. 1D ^{13}C cross sections of lignin region reveal the polymer mixing patterns 8
Supplementary Figure 7. Spin diffusion in biopolymers of all three wood samples 9
Supplementary Figure 8. ^{13}C–^{1}H dipolar order parameters of biopolymers in wood cell walls 10
Supplementary Figure 9. Intermolecular interactions of polymers in woody plants 11
Supplementary Figure 10. NMR restraints of polymer packing in secondary cell walls 12
Supplementary Figure 11. DFT structures of lignin-carbohydrate packing 13
Supplementary Figure 12. Buildup curves for water-to-polysaccharides/lignin 14
Supplementary Figure 13. Water-edited 2D ^{13}C-^{13}C correlation spectra of polysaccharides 15
Supplementary Figure 14. The ^{13}C-T_1 relaxation curves of polysaccharides and lignin 16
Supplementary Figure 15. ^{1}H-$T_{1\rho}$ relaxation curves of polysaccharides and lignin in woody plants 17
Supplementary Figure 16: Effect of freeze-drying and rehydration on hardwood eucalyptus 18
Supplementary Figure 17: Representative flow chart for resonance assignment 19
Supplementary Table 1. ^{13}C chemical shifts of mobile molecules in woods 20
Supplementary Table 2. Molar composition of polysaccharides in wood secondary cell walls 21
Supplementary Table 3. Solution NMR HSQC ^{1}H and ^{13}C chemical shifts of biopolymers 22
Supplementary Table 4. Average solution NMR chemical shifts from literature 23
Supplementary Table 5. Deconvolution of quantitative ^{13}C spectra for lignin compositional analysis 24
Supplementary Table 6. Intermolecular interactions of polymers in intact plant stems 25
Supplementary Table 7. Water-edited intensities of polysaccharide and lignin of Eucalyptus 26
Supplementary Table 8. Water-edited intensities of polymers in poplar 27
Supplementary Table 9. Water-edited intensities of polysaccharides and lignin of spruce 28
Supplementary Table 10. ^{13}C-T_1 relaxation times of lignin and polysaccharides in the three woods 29
Supplementary Table 11. ^{1}H-$T_{1\rho}$ relaxation times of lignin and polysaccharides in three samples 30
Supplementary Table 12. Parameters of 1D NMR experiments of three wood samples 31
Supplementary Table 13. Parameters of 2D NMR experiments of three wood samples 32
Supplementary Table 14. Parameters of ssNMR experiments of never-dried samples 33
Supplementary References 34

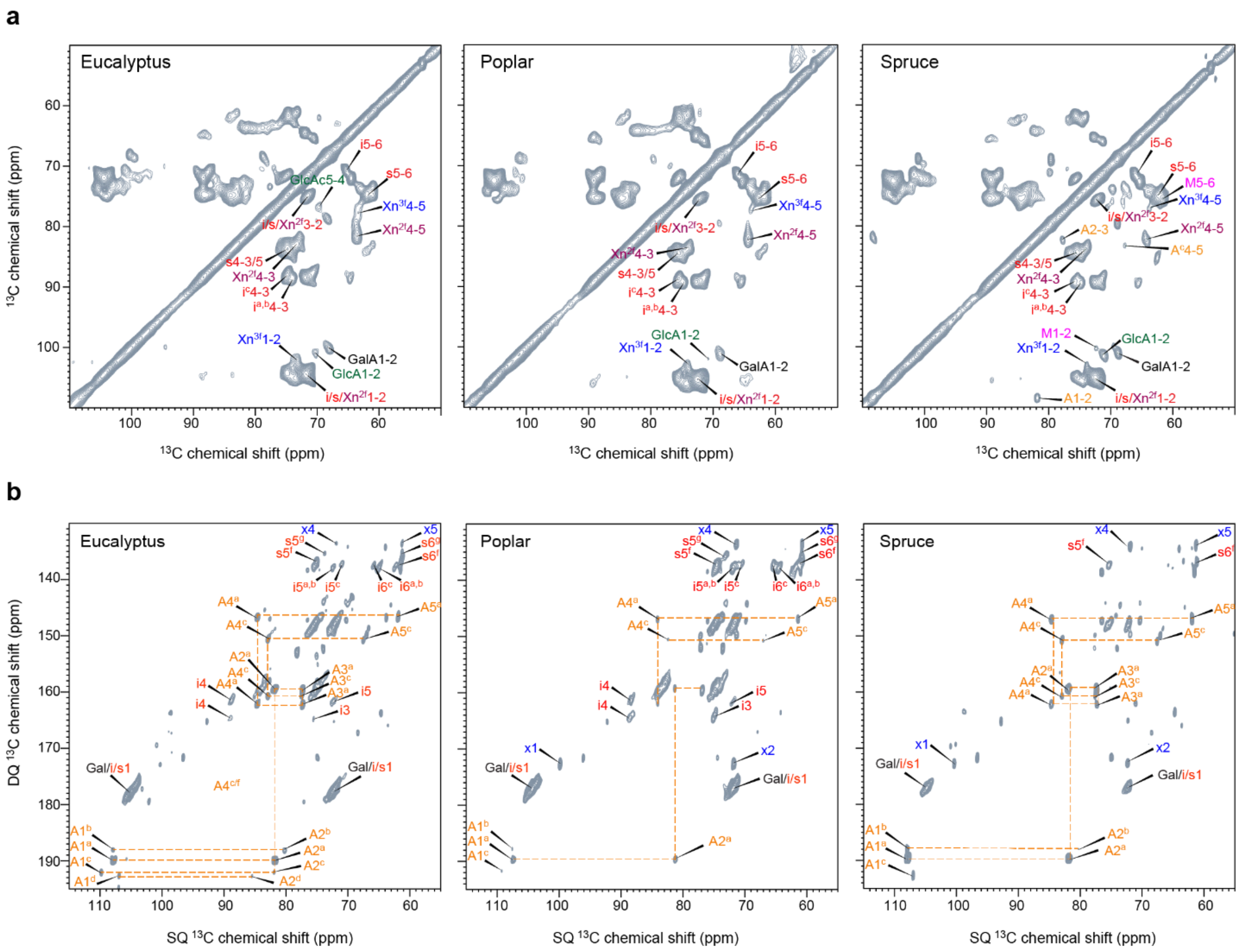

Supplementary Figure 1. 2D ^{13}C-^{13}C spectra of polysaccharides in softwood and hardwood. a, 2D ^{13}C-^{13}C RFDR spectra showing the rigid components of eucalyptus, poplar, and spruce. Uniquely, spruce has mannan and arabinose signals in the CP-based RFDR spectrum. **b**, 2D ^{13}C DP J-INADEQUATE spectra of eucalyptus, poplar, and spruce. The combination of ^{13}C DP with a short recycle delay of 2 s selectively detects the mobile molecules. Arabinose (A) signals dominate the spectra, and the four major types of arabinose are distinguished by the superscript, from A^a to A^d. The carbon connectivity of arabinose is highlighted by yellow lines. Poplar has relatively weak signals of arabinose. The ^{13}C chemical shifts are summarized in **Supplementary Data 1** and **Supplementary Table 1**.

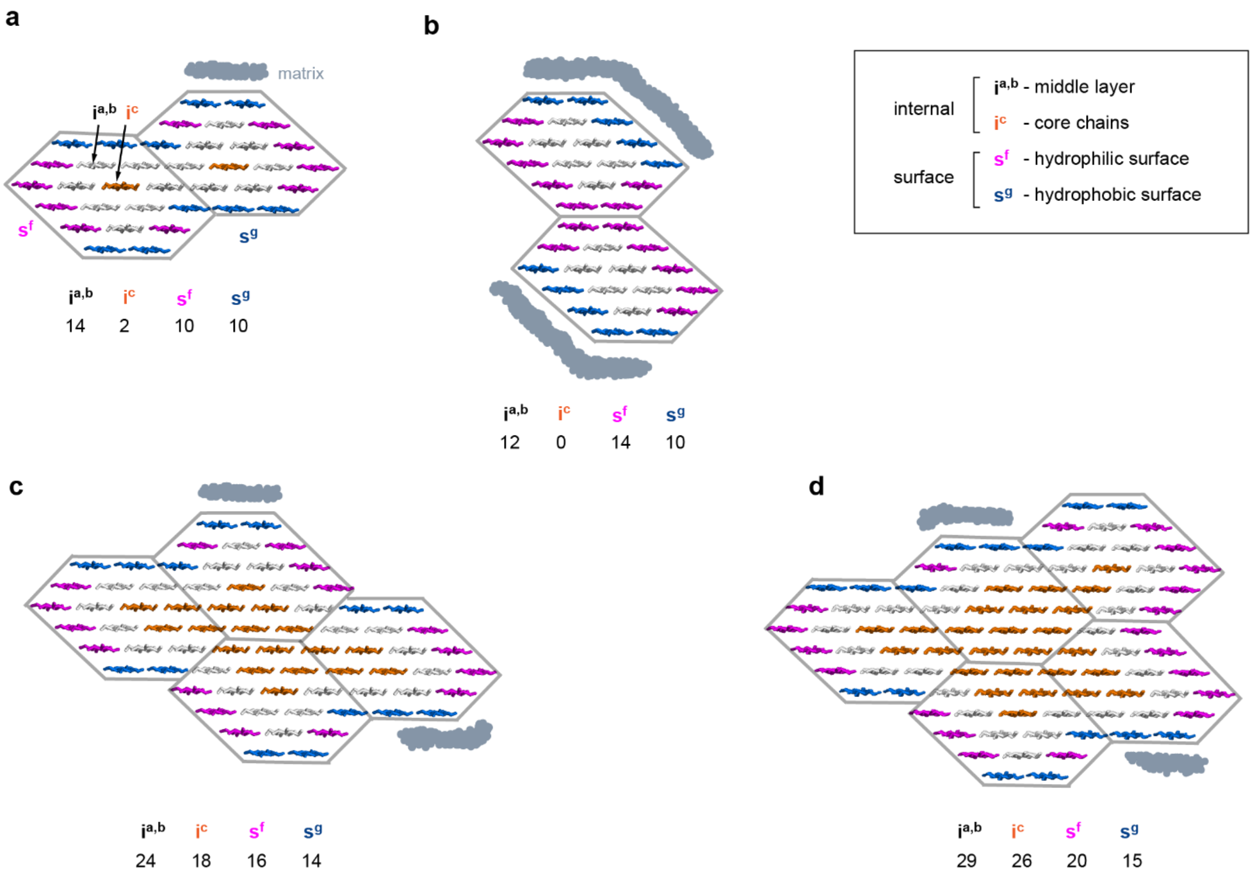

Supplementary Figure 2. Cellulose bundling models that violate the NMR constraints. The cartoon illustrations include **a and b,** two elementary microfibrils, **c,** four elementary microfibrils, and **d,** five elementary microfibrils. Each elementary microfibril is depicted to contain 18 glucan chains following the current biochemical evidence[1, 2]. The number of hydrophobic surface chains (s^g), hydrophilic surface chains (s^f), middle layer internal chains ($i^{a,b}$), and the deeply embedded core chains (i^c) are labeled[3, 4]. The matrix polymers are added to better fit the NMR constraints, but major violations still exist for all these models.

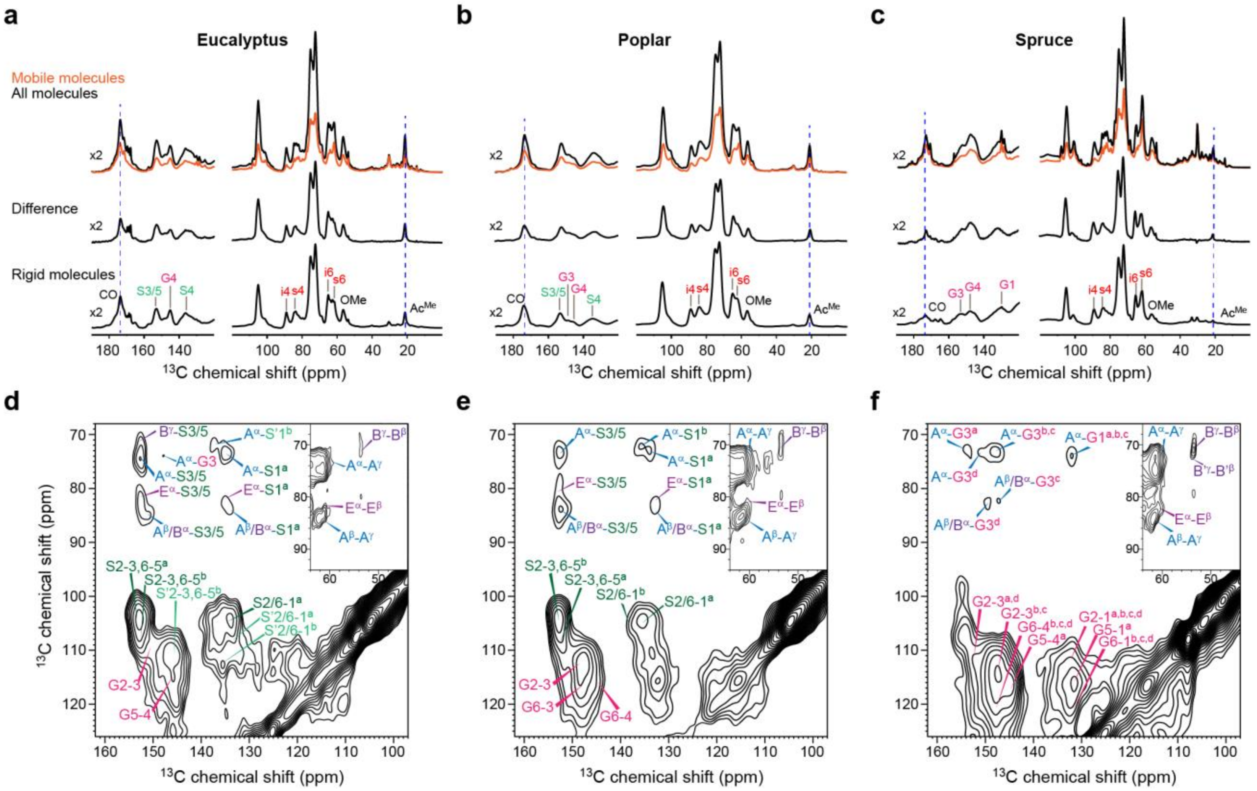

Supplementary Figure 3: Lignin distributed in the mobile and rigid phases. The top row is the quantitative-detected DP (orange), mobile molecule detected DP, rigid component detected CP spectra of **a**, eucalyptus, **b**, poplar, and **c**, spruce. All the three difference spectra of ^{13}C DP spectra with long and short recycle delays showing peaks of the rigid components, are comparable to the corresponding CP spectra. Both 2-s DP and CP spectra give well-resolved aromatic peaks, indicating dynamically heterogeneous lignin components. The bottom row is DP-PDSD spectra (1.7s recycle delay) of **d**, eucalyptus, **e**, poplar, and **f**, spruce. Abundant signals of aromatics and linkers are identified, suggesting that these linkers are also revolved at mobile phases of lignin.

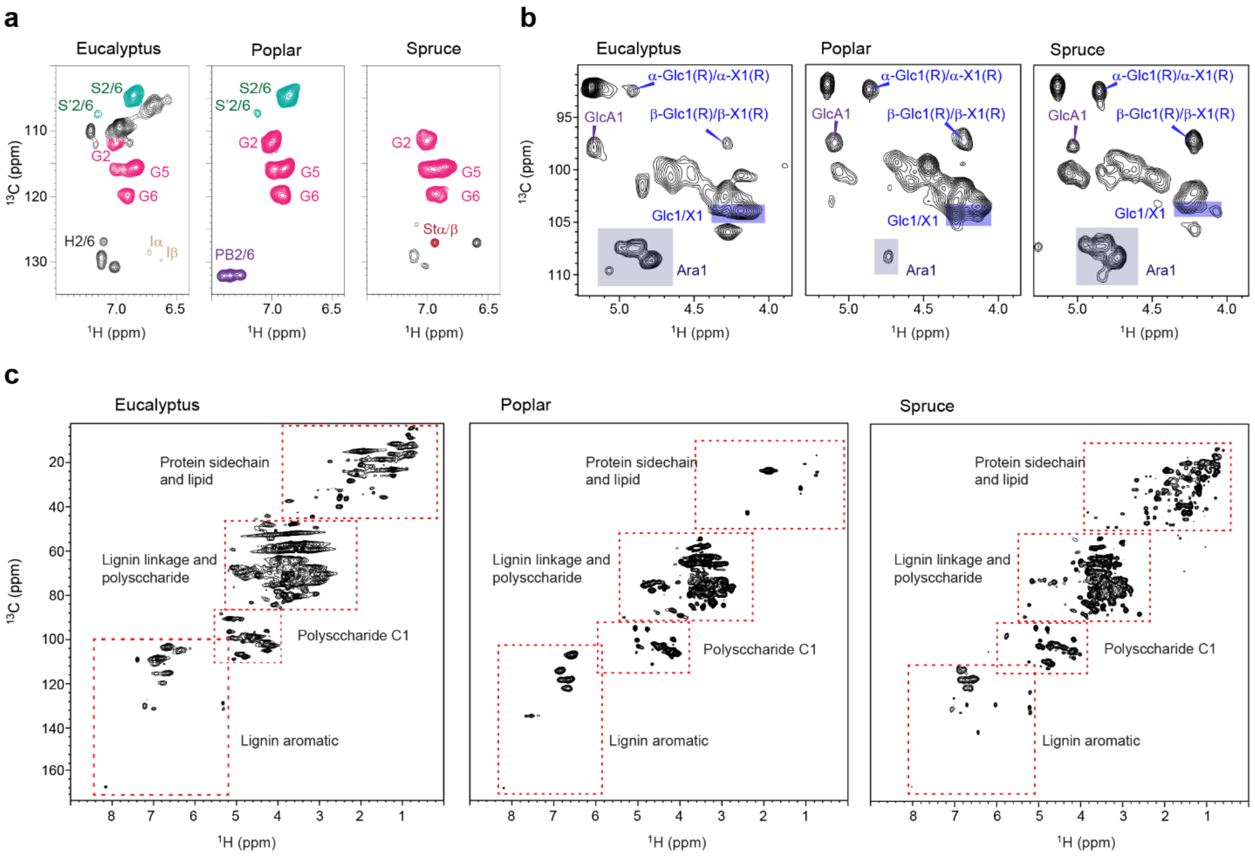

Supplementary Figure 4. Solution NMR of lignin and polysaccharides in woody stems. 2D ^{1}H-^{13}C HSQC spectra showing selected regions of **a**, mainly lignin signals and **b**, mainly polysaccharide peaks. Arabinose (Ara) is more predominant in spruce than in eucalyptus and poplar. **c,** Full HSQC spectra of the three samples. The ^{13}C and ^{1}H chemical shifts are documented in **Supplementary Table 3** and supported by a literature surveil compiled in **Supplementary Table 4**.

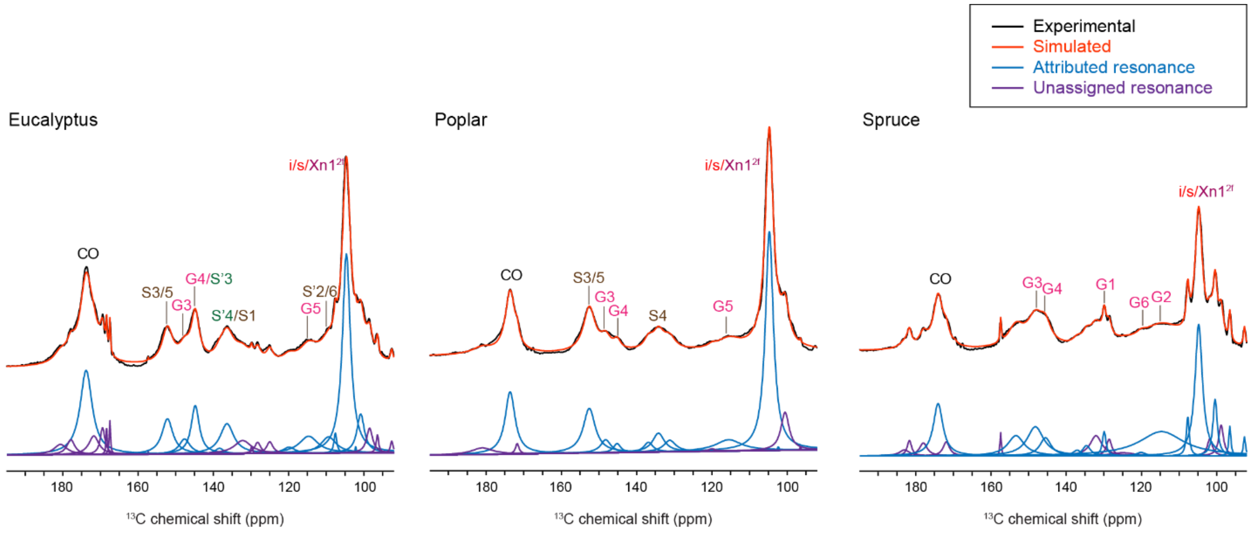

Supplementary Figure 5. Spectral deconvolution reveals the lignin composition. Deconvolution is conducted on 1D quantitative ^{13}C DP spectra collected with long recycle delays of 35-40 s. The experimentally measured spectra (black) is overlaid with the simulated spectra (red) for eucalyptus, poplar, and spruce. The bottom panels show the deconvoluted peaks. Attributed resonances are plotted in blue and uncertain resonances are in purple. Deconvolution is conducted using the DMfit software[5], using a Lorentzian model on a 92-195 ppm chemical shift window. The fitting parameters are summarized in **Supplementary Table 6**.

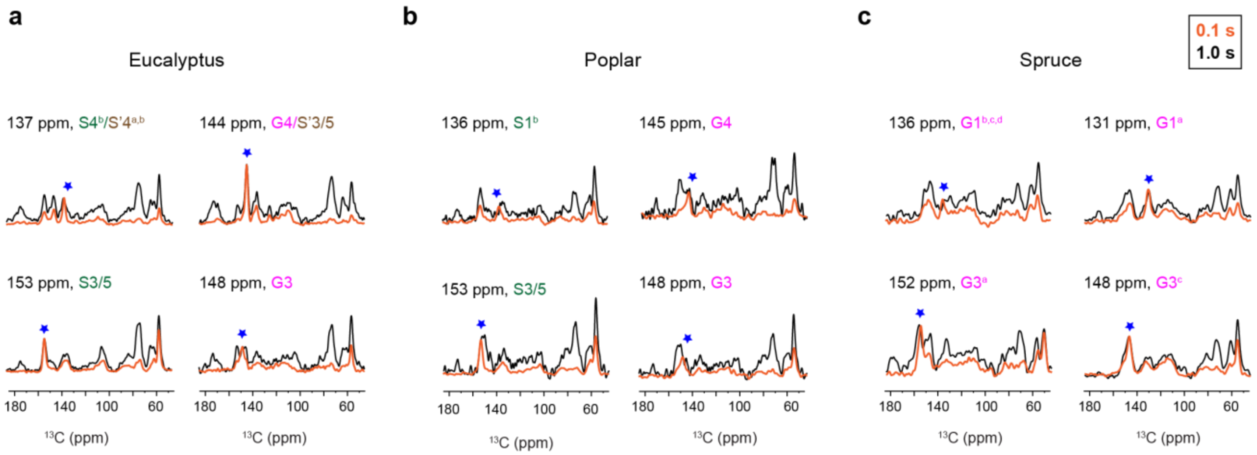

Supplementary Figure 6. 1D ^{13}C cross sections of lignin region reveal the polymer mixing patterns. Representative cross sections are obtained from dipolar gated 2D ^{13}C-^{13}C correlation spectra (**Fig. 3a**) of **a,** eucalyptus, **b,** poplar, and **c,** spruce. For eucalyptus and poplar, the first column shows the cross sections from S-lignin units and the second column shows those from G units. All the cross sections are normalized by the diagonal peaks (blue asterisks). The more similar the spectral pattern between 0.1 s and 1.0 s, the more homogeneous the polymers are mixed on the sub-nanometer scale. The spruce cross sections are more equilibrated.

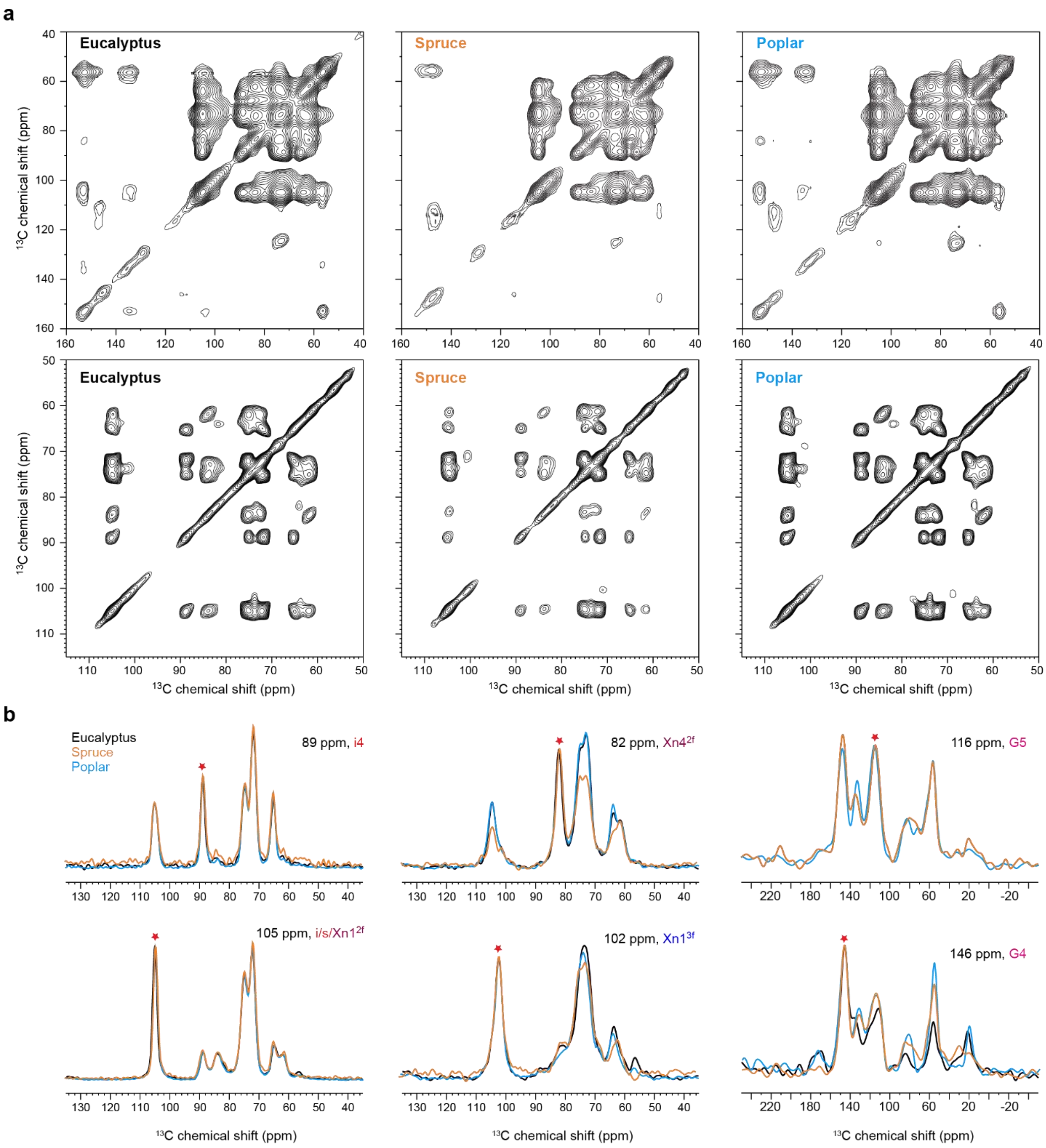

Supplementary Figure 7. Comparable spin diffusion rates of biopolymers in three wood samples. a, Aromatics regions (top row) and carbohydrates regions (second row) of 0.1s PDSD spectra of three woody plants. **b**, Representative cross peaks for cellulose, xylan, and lignin are obtained from 0.1s PDSD spectra, which are normalized by the diagonal peaks (red asterisks). The similar spectral patterns among three wood samples indicates the spin diffusion rates of lignin and carbohydrates are comparable in all three woody stems.

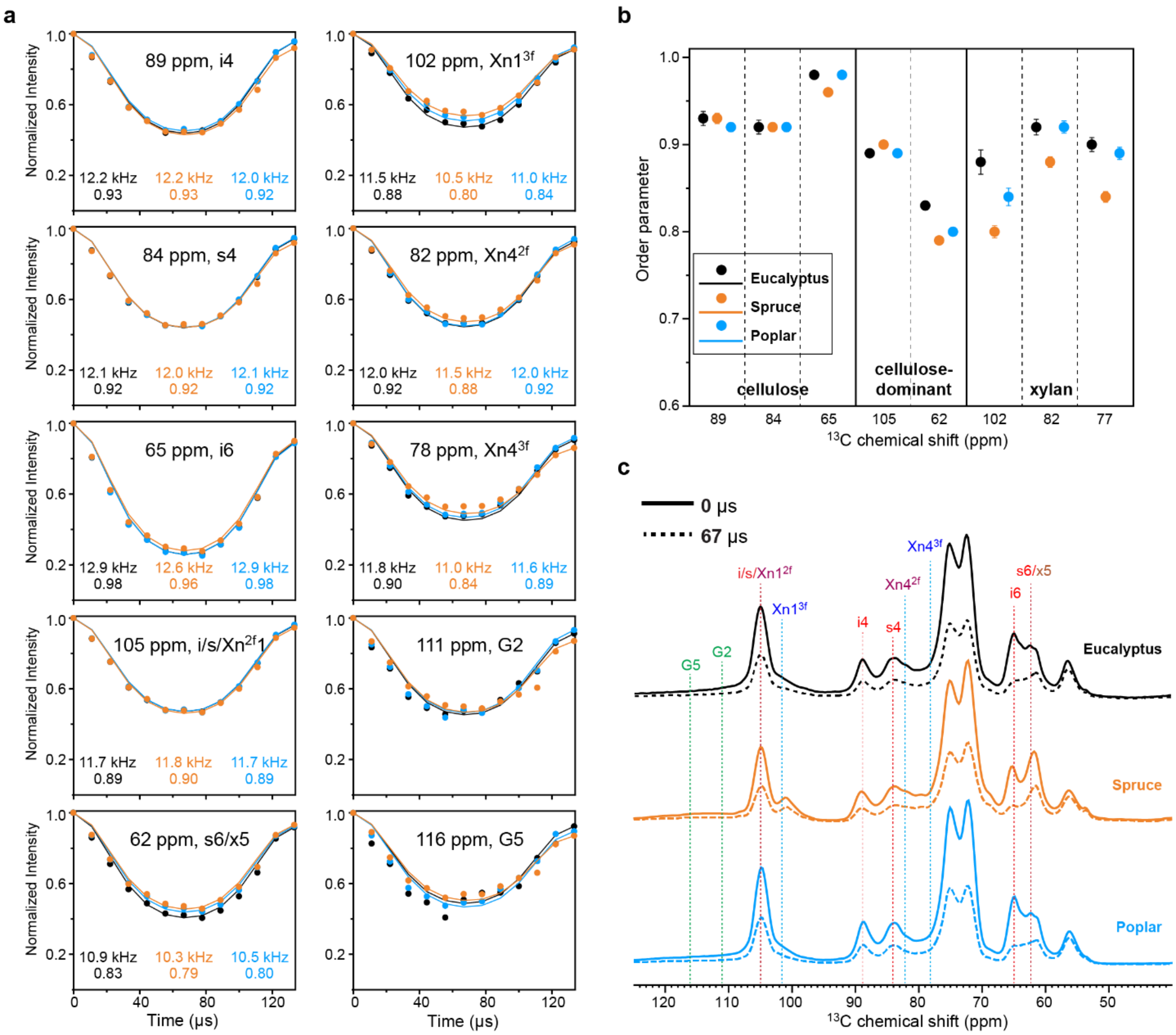

Supplementary Figure 8. ^{13}C–^{1}H dipolar order parameters of biopolymers in wood cell walls. a, Dipolar dephasing curves of carbohydrates and lignin processed from DIPSHIFT spectra. The spectra were collected under 7.5 kHz MAS with a 0.577 scaling factor for C-H bond. **b**, Summary of C–H dipolar order parameters with error bars. Spruce shows a relatively smaller order parameter for xylan. **c**, The first (0 μs dipolar dephasing) and middle (67 μs dipolar dephasing) slices of the 2D DIPSHIFT experiment imply how much the spectra are suppressed at a half rotor period. Source data are provided as a Source Data file.

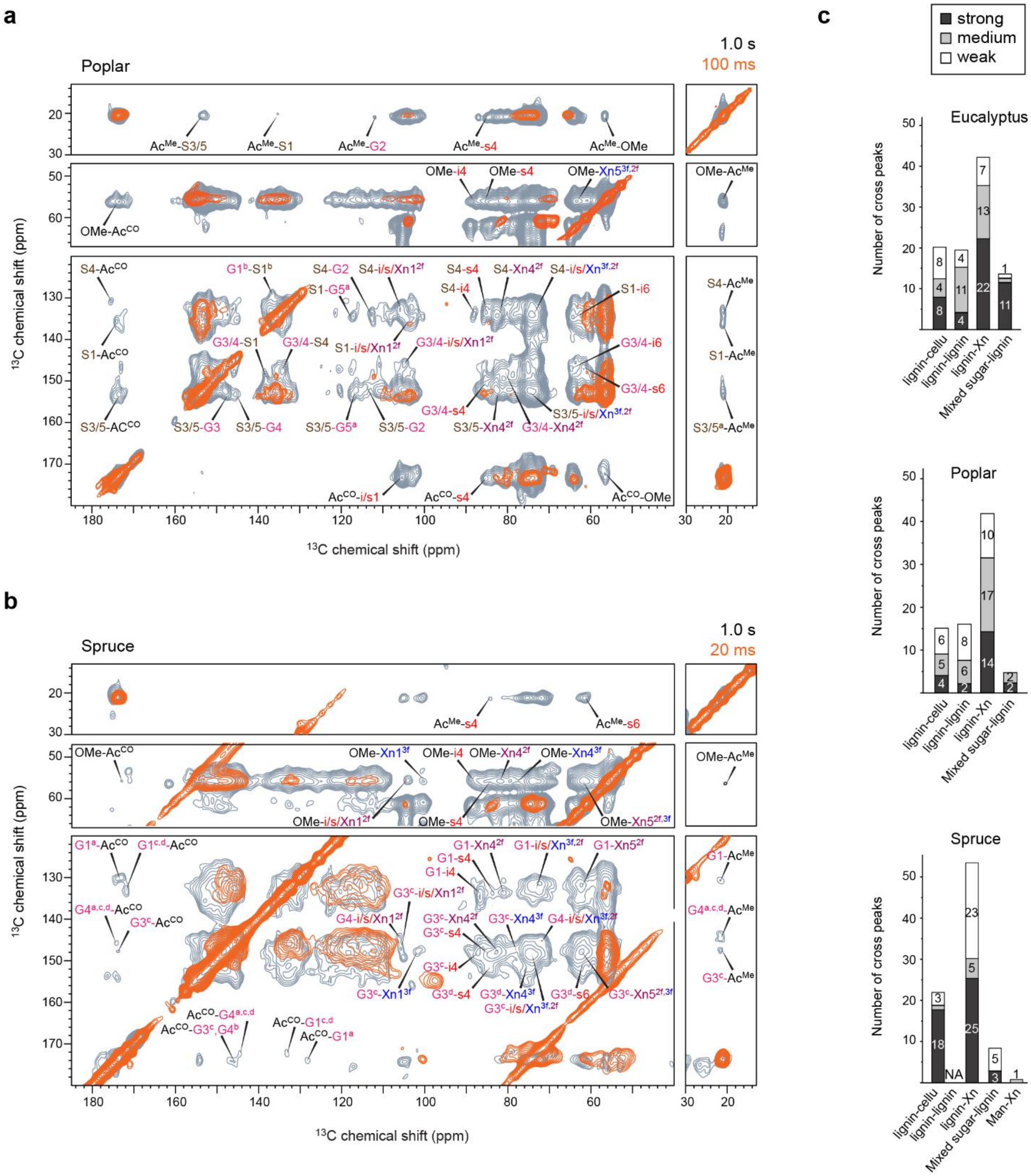

Supplementary Figure 9. Intermolecular interactions of polymers in woody plants. Dipolar-gated 2D ^{13}C-^{13}C correlation spectra measured with 1.0 s and 100 ms (poplar), 1.0 s and 20 ms (spruce) are overlaid for **a,** poplar and **b,** spruce. 100 ms (poplar) and 20 ms (spruce) spectra mainly detect the intramolecular correlations, and the 1 s spectra show many intermolecular cross peaks. Only the intermolecular cross peaks are labeled. **c**, The bar diagrams show the number of interactions between different molecules in the woods. The interactions are categorized according to the peak intensity. The details of the short-range and long-range cross peaks are summarized in **Supplementary Data 2-4**. Source data are provided as a Source Data file.

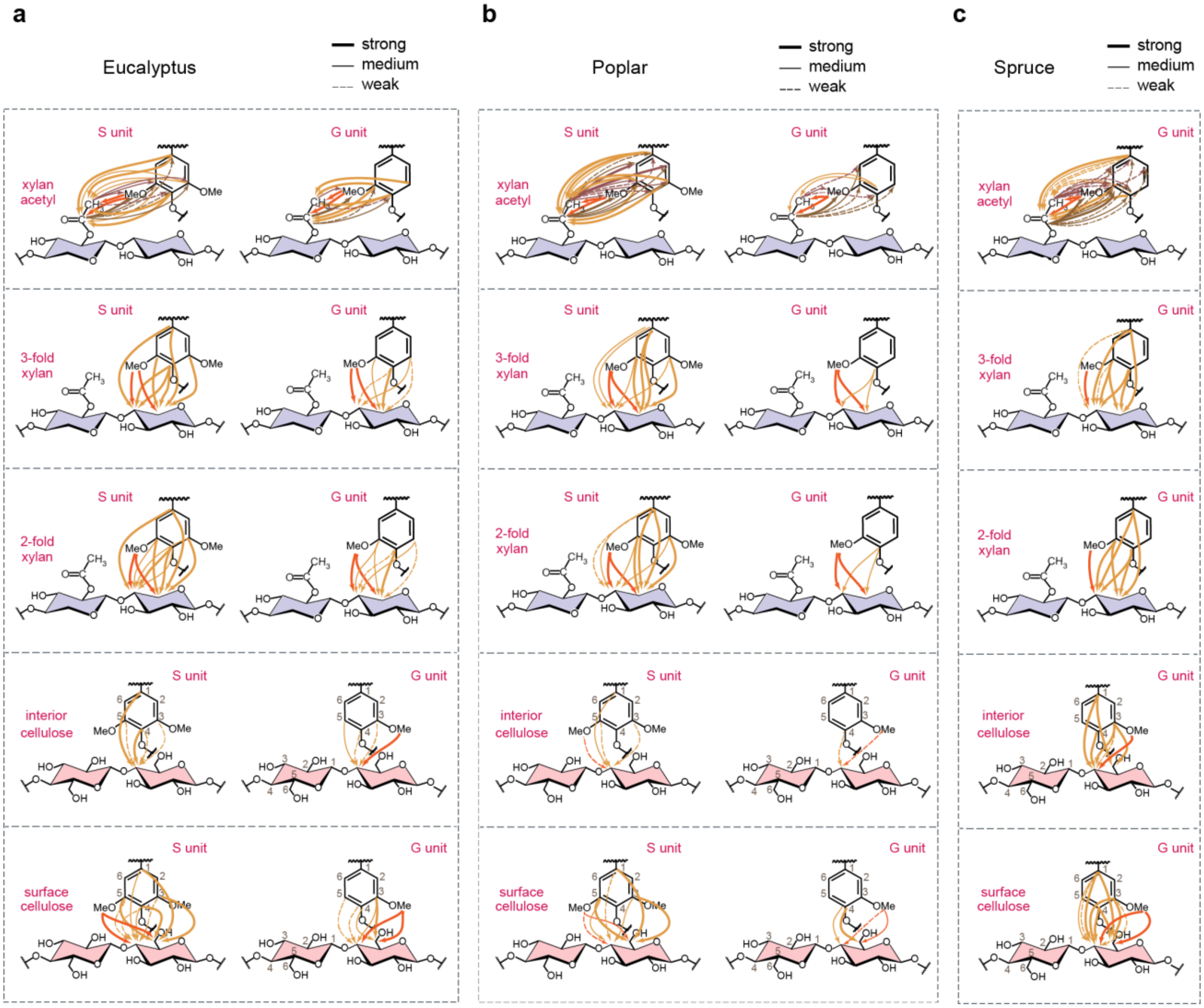

Supplementary Figure 10. NMR restraints of polymer packing in secondary cell walls. The NMR-observed restrained are presented separately for **a**, eucalyptus, **b**, popolar, and **c**, spruce. Thick lines, thin lines, and dash lines are used to represent strong, medium, and weak cross peaks observed in solid-state NMR spectra, respectively. Three major types of interactions happen between xylan acetyl and lignin (brown), between lignin methyl ether and carbohydrates (orange), and between lignin aromatics and carbohydrates (yellow). The details of these cross peaks are tabulated in **Supplementary Data 2-4** and **Supplementary Table 6**.

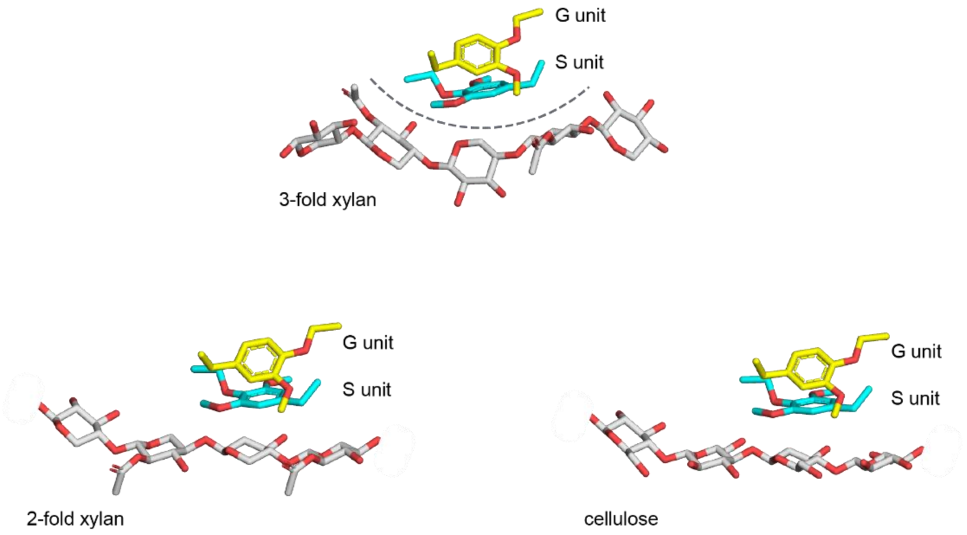

Supplementary Figure 11. DFT structures of lignin-carbohydrate packing. The structure includes the complex formed between G (yellow) or S (cyan) unit of lignin and three types of polysaccharides. The structures were adapted from an earlier study.[6]

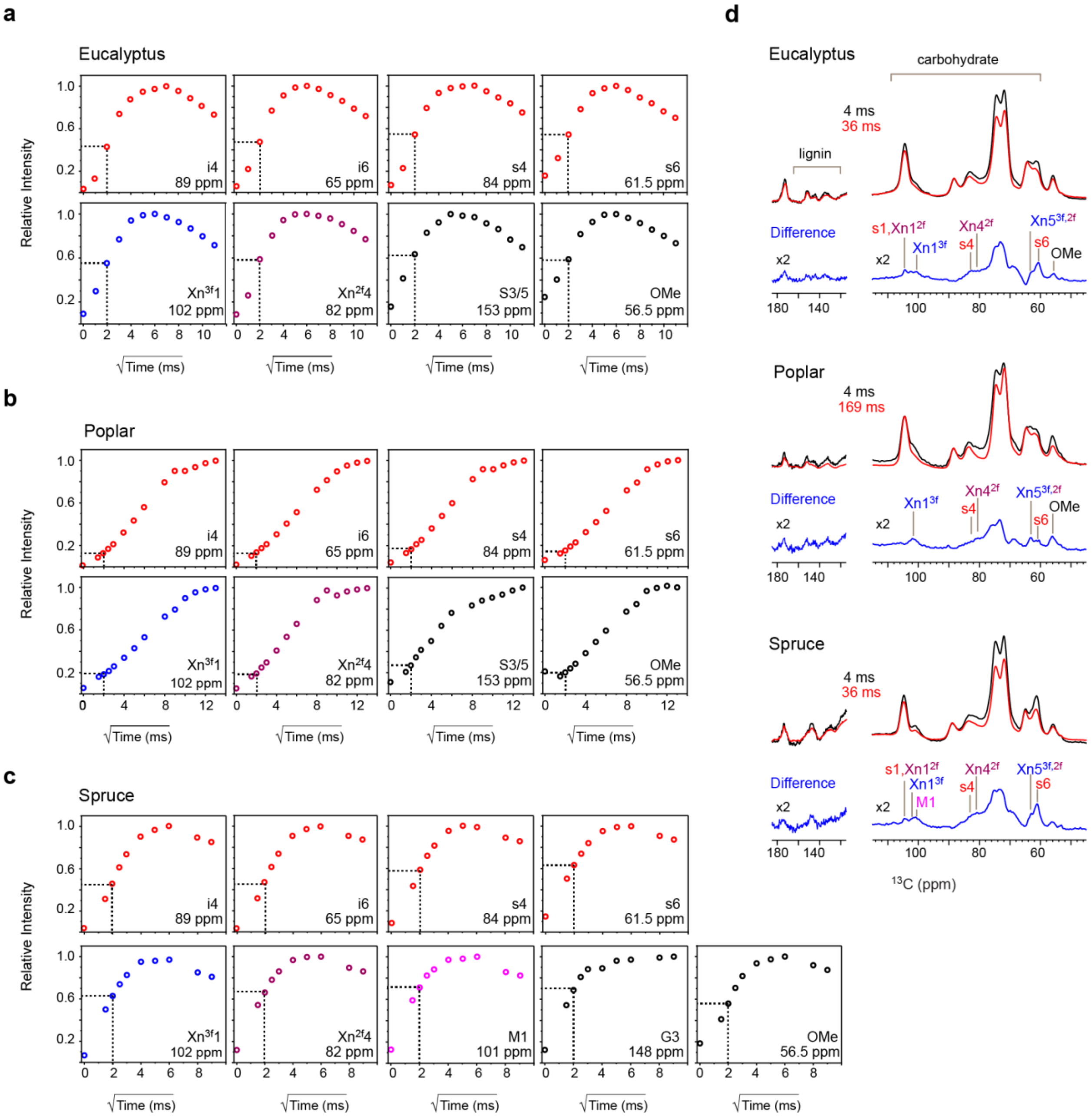

Supplementary Figure 12. Buildup curves for water-to-polysaccharides/lignin. The water ^{1}H spin diffusion curves for cellulose, xylan, and lignin for **a,** Eucalyptus, **b,** poplar and **c,** spruce. Dash lines indicate the intensities of 4-ms ^{1}H mixing. Poplar has the slowest spin diffusion from water among the three woods, with ~15-20% of the equilibrium intensity detected at 4-ms ^{1}H mixing. Eucalyptus and spruce have the fastest spin diffusion with ~45-60% of the equilibrium intensity detected at 4-ms ^{1}H mixing. **d,** Water-edited spectra measured with different mixing times. The 4-ms spectrum detects the hydrated molecules and the 36-ms (eucalyptus and spruce) and 169-ms (poplar) spectra report equilibrium intensities. The difference spectrum only shows well-hydrated molecules. Source data are provided as a Source Data file.

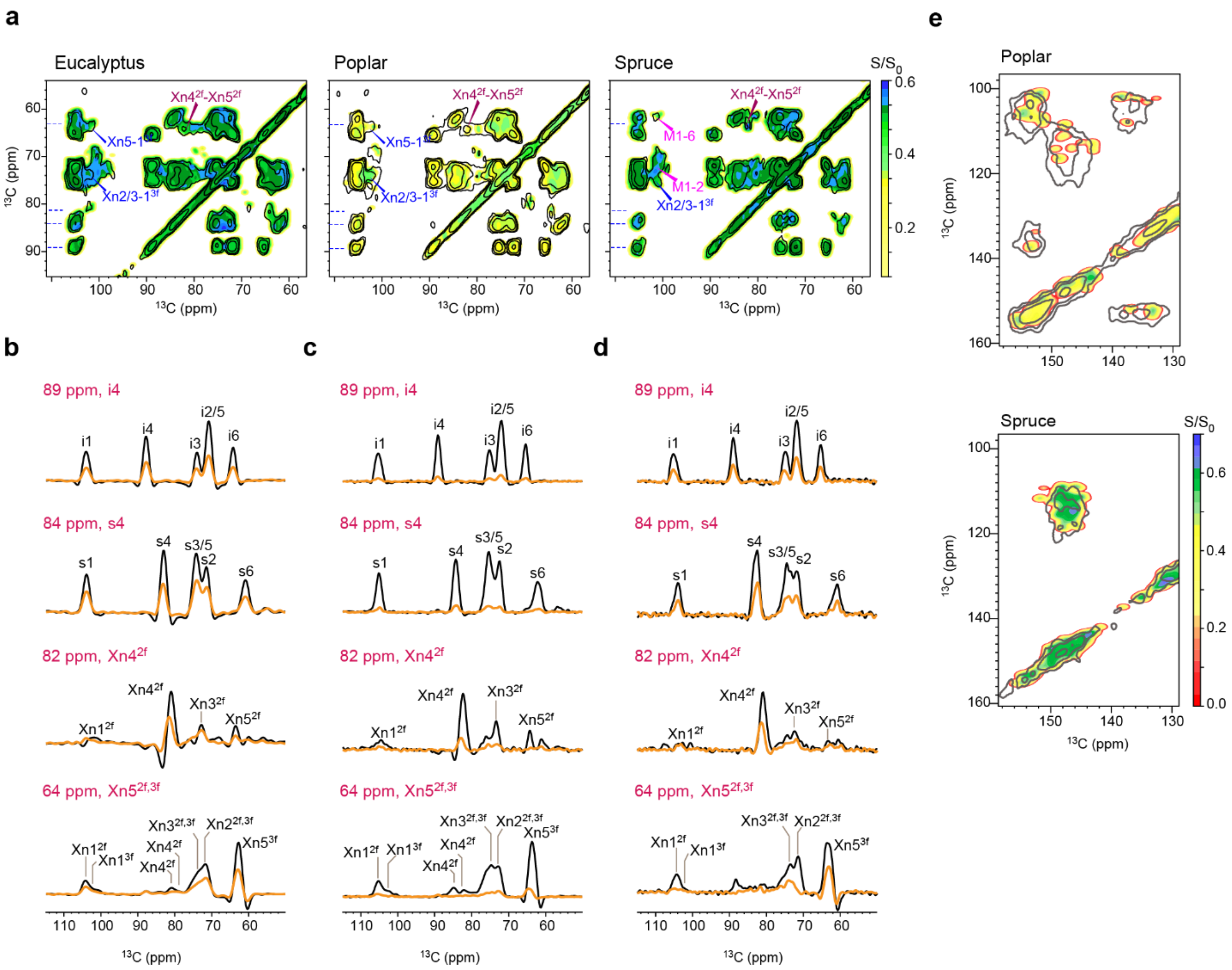

Supplementary Figure 13. Water-edited 2D ^{13}C-^{13}C correlation spectra of polysaccharides. **a**, Overlay of water-edited and equilibrium spectra showing hydration maps with S/S_0 values for eucalyptus, poplar, and spruce. The blue dash lines indicate the positions at which the ^{13}C cross sections are extracted. The representative 1D ^{13}C cross sections of cellulose (interior: i4; surface: s4) and xylan (Xn4 and Xn5) are shown for **b**, Eucalyptus, **c**, poplar, and **d**, spruce. The water-edited spectra and the equilibrium spectra are plotted in orange and black, respectively. The 3-fold and 2-fold xylans have enhanced intensity in the water-edited spectra, indicating better interactions with water molecules. **e**, Hydration maps of lignin regions with S/S_0 values for poplar (top) and spruce (bottom). Spruce is well hydrated compared to poplar as it shows higher S/S_0 values. The water-edited intensities are summarized in **Supplementary Tables 7-9**.

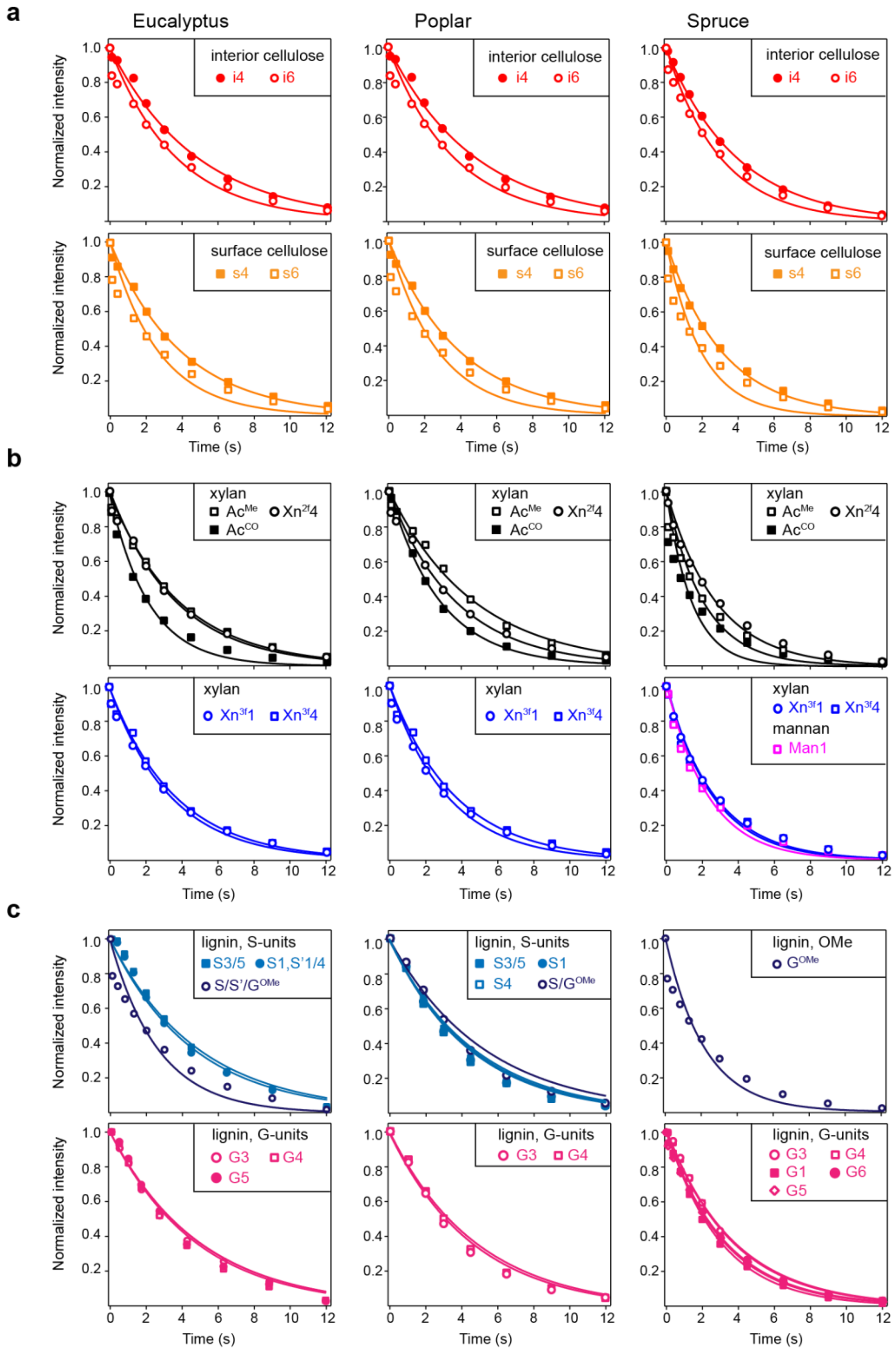

Supplementary Figure 14. The ^{13}C-T_1 relaxation curves of polysaccharides and lignin. The ^{13}C-T_1 relaxation curves of **a**, cellulose (interior and surface glucan chains), **b**, hemicellulose (2-fold and 3-fold xylan; mannan), and **c**, lignin (S and G units) are shown. The left, middle, and right columns are for eucalyptus, poplar, and spruce, respectively. The data are fitted using a single exponential equation. The fit parameters are summarized in **Supplementary Table 10**. Source data are provided as a Source Data file.

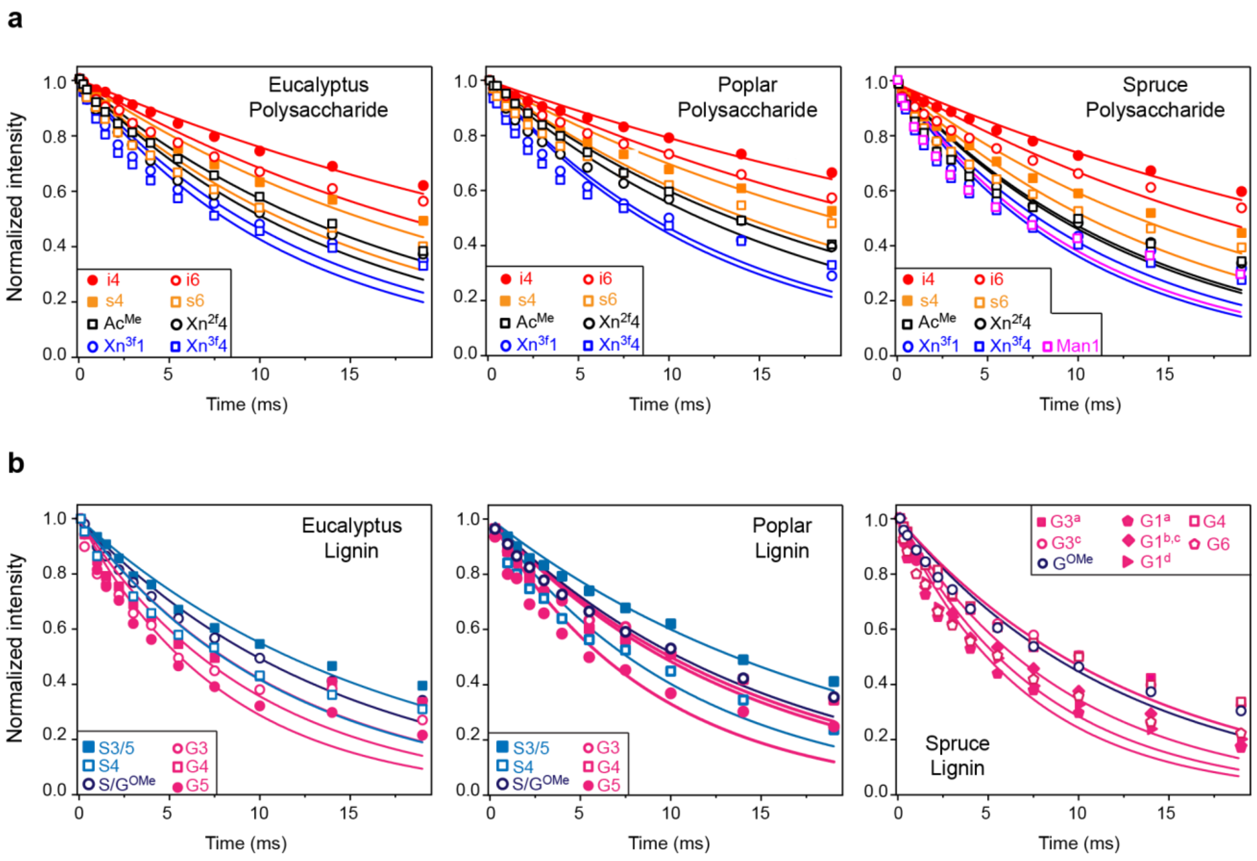

Supplementary Figure 15. ^{1}H-$T_{1\rho}$ relaxation curves of polysaccharides and lignin in woody plants. The ^{1}H-$T_{1\rho}$ relaxation curves of **a**, polysaccharides and **b**, lignin data are shown for eucalyptus, poplar, and spruce. The data are fitted using a single exponential equation. Xylan shows faster relaxation times compared to cellulose. Within lignin, the G residue has faster ^{1}H-$T_{1\rho}$ relaxation than the S unit. The fit parameters are summarized in **Supplementary Table 11**. Source data are provided as a Source Data file.

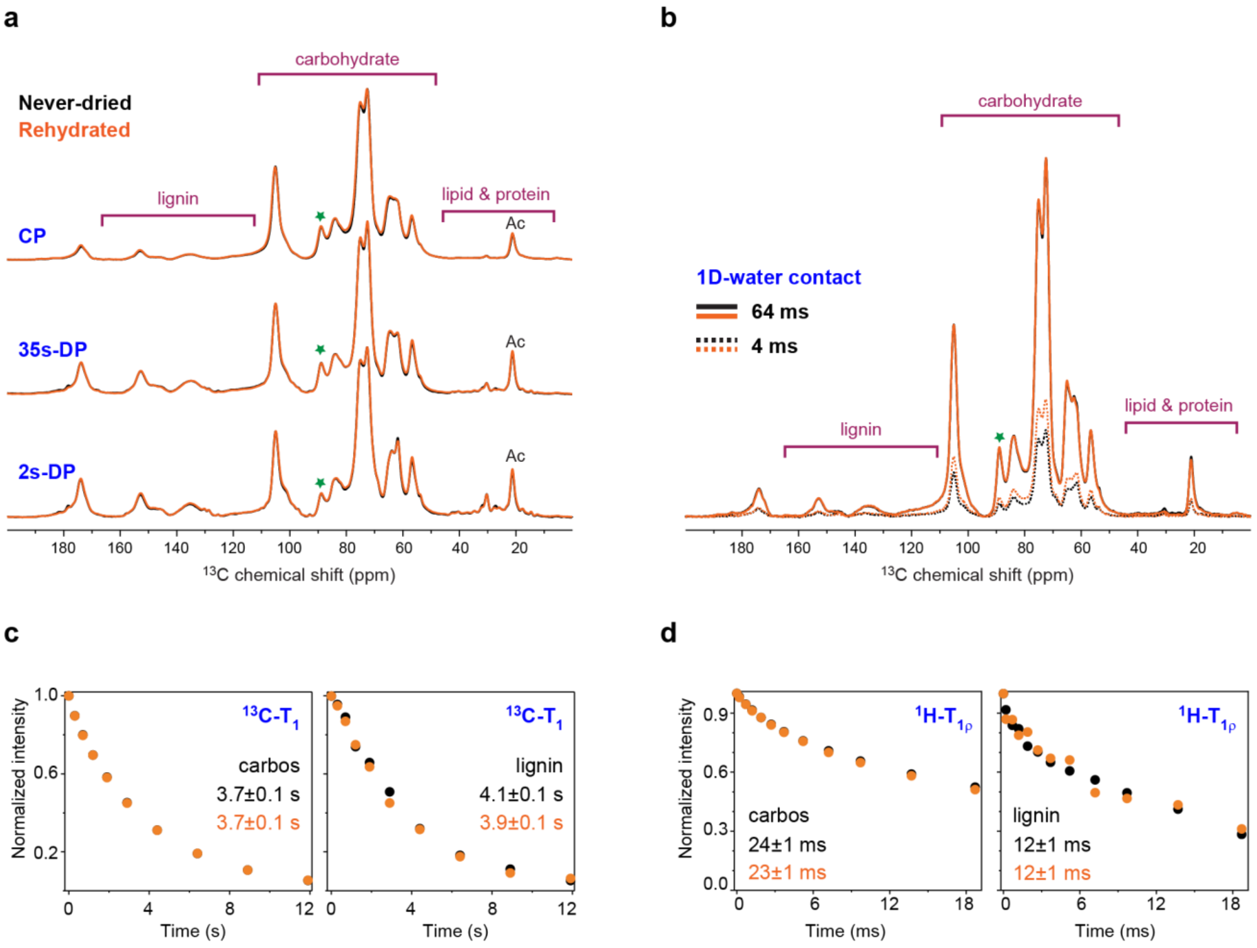

Supplementary Figure 16. Effect of freeze-drying and rehydration on hardwood eucalyptus. a, CP (rigid components selection), quantitative DP and DP (mobile components selection) normalized to i4 peak at 89 ppm. Distinct chemical shift intervals for lignin, carbohydrate, and lipid are highlighted. **b**, T_2-filtered (water-edited) 1D experiments. Signal maxima observed after 64 ms of spin diffusion, in comparison to the previous 36 ms and 26% signal difference in the absence of diffusion upon rehydration are consistent with both a sample less prone to deep hydration and a slight betterment of its surface water access after freeze-drying. **c**, Average ^{13}C T_1 relaxation curves, using an evolution of integrals of spectral regions established in panel (a). **d**, Average ^{1}H-$T_{1\rho}$ relaxation using the same two integrated intervals. Error bars are standard deviations of the fitting derived parameters. Source data are provided as a Source Data file. All these spectra and fitted relaxation parameters are very close for the fresh and rehydrated samples, demonstrating that structure and dynamics are not significantly altered by this sample preparation method. Their overall consistency with the main text eucalyptus sample also validates experimental reproducibility.

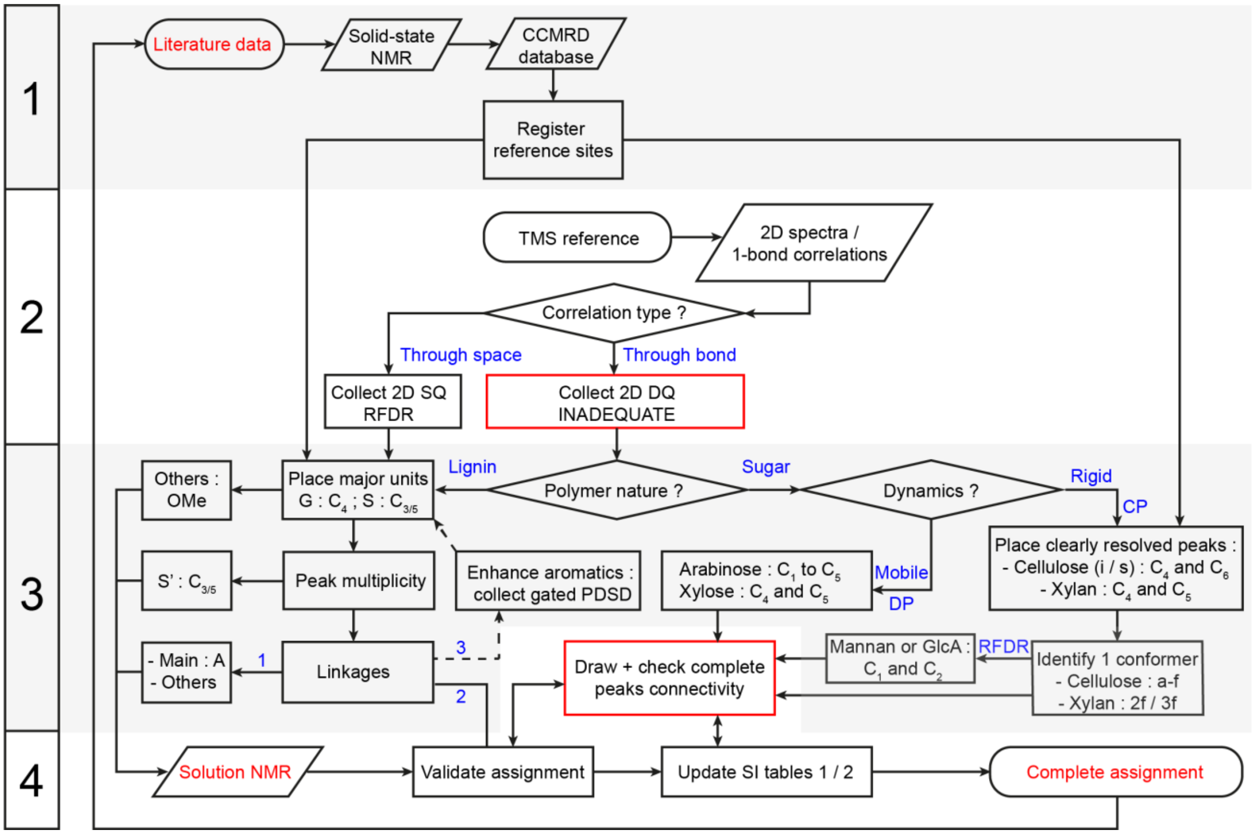

Supplementary Figure 17: Representative flow chart for resonance assignment. Shading and numbering on the left highlight the four main steps, while red coloring denotes essential sub-steps of the protocol followed for assigning chemical shifts. 1) Collection of reference chemical shifts for expected resolved peaks from solid-state NMR literature and databases. For each carbon site, we report associated references in **Supplementary Data 5**. 2) Acquisition of 2D solid-state NMR spectra. Subsequent assignments rely primarily on the resolution provided by the DQ dimension of INADEQUATE spectra. 3) Sequential assignment (boxes from right to left), from well-established cellulose and xylan conformers to mobile primary cell wall polysaccharides, ending with lignin and its linkages. For each polymer, we indicate a well-established site from which assignment can be easily initiated, while less resolved sites are either identified via cross-peaks connections or from literature reported values. 4) Safeguarding steps taken to provide accountability in the final assignments. Particularly for the complex lignin assignment, a good match is demanded with solution NMR spectra, as summarized in **Supplementary Table 3 and Supplementary Data 5.**

Supplementary Table 1. ^{13}C chemical shifts of mobile molecules in woods. The mobile components are identified from ^{13}C DP-based INADEQUATE spectra measured with short recycle delays of 2 s. Superscripts are used to denote different allomorphs. Not applicable (/). Unidentified (-).

	**carbohydrate**	C1	C2	C3	C4	C5	C6	Plants
A^a	arabinose	107.8	81.8	77.2	84.6	61.8	/	All samples
A^b		107.8	80.1	-	-	-	/	All samples
A^c		109.7	81.8	77.4	82.9	67.6	/	All samples
A^d		106.9	85.3	-	-	-	/	Eucalyptus
Gal	galactose	104.9	72.3	-	-	-	-	All samples
α-Glc	α-glucose	92.7	-	-	-	-	-	Spruce
β-Glc	β-glucose	96.6		-	-	-	-	Spruce
x	xylose in XyG	100.3	72.3	-	71.9	61.3	/	All samples
$i^{a,b}$	cellulose	-	-	75.5	88.9	72.4	64.9	Eucalyptus
i^c		-	-	75.5	88.9	70.9	65.8	Eucalyptus
$i^{a,b}$		-	-	75.5	88.9	72.4	64.9	Poplar
i^c		-	-	75.5	88.9	70.9	65.8	Poplar
s^f		-	-	-	-	75.0	61.4	All samples
s^g		-	-	-	-	73.7	61.2	Eucalyptus
s^g		-	-	-	-	73.7	61.2	Poplar
	**lignin**	C1	C2	C3	C4	C5	C6	Plants
G^a	guaiacyl	136.3	-	144.1	144.1	-	-	Eucalyptus
G^b		136.3	-	145.2	145.2	-	-	
S^a	syringyl	136.7	-	152.9	-	152.9	-	
S^b		135.5	-	151.7	-	151.7	-	
G	guaiacyl	130.1	-	147.8	146.4	-	-	Poplar
S^a	syringyl	-	-	152.6	-	152.6	-	
S^b		-	-	151.9	-	151.9	-	
G	guaiacyl	-	-	148.9	144.7	-	-	Spruce
G		-	-	152.3	-	152.3	-	

Supplementary Table 2. Molar composition of polysaccharides in wood secondary cell walls. For each plant, the composition of cellulose, xylan, mannan, and the primary cell wall component xyloglucan (XyG) is given. Cellulose contains interior glucan chains ($i^{a,b}$: middle layer; i^{c}: embedded core chains) and surface glucan chains (s^{f}: hydrophilic surface; s^{g}: hydrophobic surface). Xylan contains the backbone (Xn^{2f}: two-fold xylan; Xn^{3f}: three-fold xylan; Xn: mixed conformation) and sidechains (GlcA and/or Ara). For Mannan, only the mannose residues (M: unacetylated; M^{Ac}: acetylated) are resolved, while the actual fraction of mannan should be higher than the value reported here due to the presence of Glc residues in the backbone. The sidechain α-xylose (x) is used to denote xyloglucan.

Eucalyptus

Cellulose 74.0%				Xylan 25.5%					Mannan -		XyG 0.5%
interior chains 38.2%		surface chains 35.8%		backbone 16.8%			sidechains 8.7%		- -		
$i^{a,b}$	i^{c}	s^{f}	s^{g}	Xn^{2f}	Xn	Xn^{3f}	GlcA	Ara	M	M^{Ac}	x
28.3%	9.8%	21.2%	14.6%	10.0%	2.0%	4.9%	8.7%	-	-	-	0.5%

Poplar

Cellulose 81.4%				Xylan 18.0%					Mannan -		XyG 0.6%
interior chains 45.0%		surface chains 36.4%		backbone 13.3%			sidechains 4.8%		-		
$i^{a,b}$	i^{c}	s^{f}	s^{g}	Xn^{2f}	Xn	Xn^{3f}	GlcA	Ara	M	M^{Ac}	x
32.7%	12.3%	21.9%	14.6%	7.9%	1.6%	3.8%	4.8%	-	-	-	0.6%

Spruce

Cellulose 75.8%				Xylan 11.8%					Mannan 10.5%		XyG 1.9%
interior chains 37.1%		surface chains 38.7%		backbone 10.4%			sidechains 1.5%				
$i^{a,b}$	i^{c}	s^{f}	s^{g}	Xn^{2f}	Xn	Xn^{3f}	GlcA	Ara	M	M^{Ac}	x
28.1%	8.9%	23.6%	15.2%	6.9%	-	3.5%	0.5%	1.0%	6.2%	4.3%	1.9%

The area of the following well-resolved peak pairs in CP J-INADEQUATE spectra are used:
$i^{a,b}$: the average of $i5^{a,b}$ - $i6^{a,b}$, $i4^{a,b}$ - $i5^{a,b}$, and $i3^{a,b}$ - $i4^{a,b}$.
i^{c} : the average of $i5^{c}$ - $i6^{c}$, $i4^{c}$ - $i5^{c}$, and $i3^{c}$ - $i4^{c}$.
s^{f} : the average of $s5^{f}$ - $s6^{f}$, $s4^{f}$ - $i5/3^{f}$. The integration of $s4^{f}$ - $i5/3^{f}$ has been doubled because 2 peak pairs are included (carbon 4-carbon 5 and carbon 4-carbon 3).
s^{g} : the average of $s5^{g}$ - $s6^{g}$, $s4^{g}$ - $i5/3^{g}$. The integration of $s4^{g}$ - $i5/3^{g}$ has been doubled because 2 peak pairs are included (carbon 4-carbon 5 and carbon 4-carbon 3).
Xn^{2f}: the average of $Xn4^{2f}$ and $Xn5^{2f}$.
Xn: the average of Xn4 and Xn5.
Xn^{3f}: the average of $Xn4^{3f}$ and $Xn5^{3f}$.
GlcA: the average of GlcA1 and GlcA2. The value reported are the sum of $GlcA^{a}$, $GlcA^{b}$ and $GlcA^{c}$.
Ara: the average of A1 and A2.
M: the average of M1 and M2.
M^{Ac}, the average of $M1^{Ac}$ and $M2^{Ac}$.
x: the average of x4 and x5.

Supplementary Table 3. Solution NMR HSQC ^{1}H and ^{13}C chemical shifts of lignin aromatics and linkages. Unidentified or unresolved (-).

		Eucalyptus		Poplar		Spruce	
		^{13}C (ppm)	^{1}H (ppm)	^{13}C (ppm)	^{1}H (ppm)	^{13}C (ppm)	^{1}H (ppm)
G2	guaiacyl	111.9	6.99	111.6	7.00	111.6	7.0
G5	guaiacyl	115.5	6.67	115.7	6.78	114.9	6.77
		& 115.8	& 6.97	& 115.8	& 6.97	& 115.9	& 6.93
G6	guaiacyl	119.9	6.99	119.8	6.83	119.7	6.86
G’2	oxidized guaiacyl	112.2	7.53	112.4	7.53	112.2	7.51
G’6	oxidized guaiacyl	124.2	7.16	123.8	7.60	123.6	7.57
S2/6	syringyl	104.3	6.71	104.6	6.73	-	-
S’2/6	oxidized syringyl	107.3	7.32	107.2	7.24	-	-
			& 7.12		& 7.09		
H2/6		126.9	7.20	128.6	7.22	-	-
Aα (G)	β-O-4’	71.8	4.65	71.8	4.65	71.9	4.77
Aα (S)	β-O-4’	72.6	4.88	72.6	4.88		
Aβ(G)	β-O-4’	84.5	4.31	84.6	4.31	84.6	4.31
Aβ(S)	β-O-4’	86.7	4.14	86.7	4.14		
Aγ	β-O-4’	60.5	3.42	-	-	-	-
			& 3.67				
Bα	β-β’ (resinol)	85.8	4.68	84.6	4.76	85.6	4.65
Bβ	β-β’ (resinol)	53.8	3.14	54.3	3.10	53.9	3.14
Bγ	β-β’ (resinol)	71.7	3.88	70.5	3.73	70.3	3.77
			& 4.20				
Cα	β-5’ (phenylcoumaran)	87.7	5.49	87.7	5.49	87.6	5.49
Cβ	β-5’ (phenylcoumaran)	53.8	3.14	54.1	3.49	54.2	3.42
Cγ	β-5’ (phenylcoumaran)	62.7	3.44	-	-	-	-
			& 3.59				
Dα	5-5’ (dibenzodioxocin)	83.8	4.90	-	-	-	-
Dβ	5-5’ (dibenzodioxocin)	85.6	3.91	85.6	3.92	84.9	3.88
Eα	β-1’ (spirodienone)	81.8	5.07	81.9	5.08	83.7	5.02
Eα’	β-1’ (spirodienone)	86.9	4.41	87.0	4.42	-	-
Eβ	β-1’ (spirodienone)	60.4	2.79	60.7	2.80	61.8	2.8
Eβ’	β-1’ (spirodienone)	-	-	78.7	4.14	-	-
E2/2’	β-1’ (spirodienone)	114.9	6.24	114.2	6.23	114.3	6.21
E6/6’	β-1’ (spirodienone)	119.5	6.12	119.2	6.15	119.5	6.11
Iα	cinnamyl alcohol	128.3	6.46	129.0	6.45	129.5	6.48
Iβ	cinnamyl alcohol	129.7	6.25	129.5	6.27	129.1	6.32
Iγ	cinnamyl alcohol	62.4	4.11	62.3	4.12	62.5	4.13
Jα	cinnamaldehyde	154.5	7.65	154.2	7.64	154.4	7.66
Jβ	cinnamaldehyde	126.7	6.81	127.1	6.80	127.4	6.88
J6	cinnamaldehyde	122.4	7.21	123.9	7.22	124.3	7.20
PB2/6	*p*-hydroxybenzoate	-	-	132.1	7.70	-	-
Stα/β	stilbene	-	-	-	-	127.1	6.88

Supplementary Table 4. Average solution NMR chemical shifts of lignin from literature. The reference (Ref.) for each chemical shift is provided. Not detected (-).

[a] May also includes Aβ(G/H)

	Eucalyptus			Poplar			Spruce		
	^{13}C (ppm)	^{1}H (ppm)	Ref.	^{13}C (ppm)	^{1}H (ppm)	ref	^{13}C (ppm)	^{1}H (ppm)	ref
G2	111.3	6.98	7-11	111.1	6.98	12-16	110.3	6.94	17, 18
G5	115.2	6.72	7 / 9-11	115.0	6.80	12-16	115.2	6.85	17, 18
		& 6.94	7 / 9, 10	-	-	-	-	-	-
G6	119.3	6.80	7-11	119.1	6.8	12-16	118.9	6.83	17, 18
G’2	112.4	7.50	9	111.4	7.51	12	-	-	9 / 12
G’6	124.0	7.00	9	123.3	7.60	12	-	-	12
S2/6	104.2	6.99	7-11	103.8	6.67	12-16	-	-	-
S’2/6	106.5	7.31	7-11	106.1	7.22	12-16	-	-	-
H2/6	-	-	-	127.9	7.19	12	-	-	-
Aα(G)	71.6	4.74	7 / 9, 10	71.5	4.75	13	71.1	4.75	17, 19
Aα (S)	72.2	4.87	7-11	71.9	4.83	13-16	-	-	17, 19
Aβ(G)	83.9[(1)]	4.29	7-11	83.7 [a]	4.29	12, 13 / 16	84.0	4.29	17, 19
Aβ(S)	86.4	4.09	7-11	85.8	4.13	12, 13 / 15, 16	-	-	17, 19
Aγ	59.8	3.39	7-9/ 11	59.9	3.45	12, 13 / 15	59.9	3.24	17, 19
		& 3.69	7-9/ 11			12, 13		& 3.61	17, 19
Bα	85.2	4.66	7-11	85.0	4.64	12-16	85.0	4.65	17, 19
Bβ	53.7	3.08	7-9/ 11	53.6	3.07	12, 13 / 15	53.6	3.07	17, 19
Bγ	71.4	3.82	7-9/ 11	70.6	3.81	12, 13	70.9	3.74	17, 19
Cα	87.1	5.46	7-11	87.0	5.47	12-16	87.0	5.47	17, 19
Cβ	53.5	3.45	7-9/ 11	53.2	3.48	12-14	53.1	3.46	17, 19
Cγ	62.7	3.67	7, 8/ 11	62.5	3.74	12, 13	62.8	3.72	17, 19
Dα	83.6	4.83	7	81.4	4.7	13	83.3	4.84	17, 19
Dβ	85.9	3.88	7	-	-	-	85.4	3.88	17, 19
Eα	81.4	5.09	7, 8	81.2	5.07	12	81.7	5.03	19
Eα’	86.6	4.39	7	-	-	-	-	-	-
Eβ	60.0	2.74	7, 8	59.7	2.77	12	59.3	2.77	19
Eβ’	79.5	4.11	7, 8	79.5	4.12	12	-	-	-
E2/2’	112.4	6.26	7, 8	113.3	6.22	12	-	-	7, 8 / 12
E6/6’	119.1	6.07	7, 8	118.9	6.07	12	-	-	7, 8 / 12
Iα	-	-	-	128.4	6.44	12	-	-	-
Iβ	-	-	-	128.2	6.25	12	-	-	-
Iγ	61.7	4.09	7-9	61.4	4.10	12	61.5	4.08	17, 19
Jα	-	-	-	153.5	7.60	12	153.7	7.61	17
Jβ	-	-	-	126.1	6.76	12	126.3	6.77	17
J6	-	-	-	-	-	-	123.2	7.22	17
PB2/6	-	-	-	131.1	7.66	12-16	-	-	17
Stα/β	126	7.0	11	-	-	-	128.3	7.12	17

Supplementary Table 5. Spectral deconvolution of quantitative ^{13}C spectra for lignin compositional analysis. The attributed chemical shifts are labeled. Unidentified (-).

Eucalyptus

Chemical shift [ppm]	Ampli-tude	Width [ppm]	Integral [%]	Carbon
180.6	117	4.2	2.2	-
177.9	170	2.6	1.9	-
173.8	956	3.9	16.2	Ac^{CO}
171.8	211	3.2	3.0	-
169.4	306	1.5	2.0	-
168.4	314	0.4	0.5	-
167.5	385	0.4	0.7	-
157.3	36	0.1	0.02	-
152.2	401	3.5	6.2	S3/5
147.7	171	3.4	2.6	G3
144.8	550	2.6	6.3	G4/S'3
138.4	63	2.6	0.7	$S'^{b}4$
136.4	341	4.4	6.6	$S1/S'^{a}4$
132.2	149	5.5	3.6	-
129.8	79	0.6	0.2	-
128.2	128	1.9	1.1	-
125.0	133	2.0	1.2	-
119.9	70	3.8	1.2	
114.7	195	6.0	5.2	G5
109.4	185	4.9	4.0	S'2/6
107.6	229	0.9	0.9	A1
104.6	2261	2.6	25.5	$i/s/Xn1^{2f}$
102.2	75	0.3	0.1	$Xn1^{3f}$
100.8	444	2.4	4.6	Man1
98.5	281	1.9	2.4	-
96.4	209	0.8	0.7	-
92.6	135	0.8	0.5	-

Spruce

Chemical shift [ppm]	Ampli-tude	Width [ppm]	Integral [%]	Carbon
183.0	96	3.3	1.2	
181.7	248	1.4	1.3	
178.0	206	2.5	1.9	
174.1	836	3.1	9.5	Ac^{CO}
171.9	224	2.1	1.7	
169.5	36	1.1	0.1	
157.4	375	0.4	0.6	
153.4	323	5.3	6.3	
148.2	466	5.3	9.2	$G^{a}3/4$
145.5	289	3.6	3.8	$G^{b}3/4$
137.1	89	2.8	0.9	$G^{b}1$
134.6	163	2.6	1.6	$G^{c/d}1$
132.1	322	3.8	4.5	
129.9	389	1.2	1.7	$G^{a}1$
128.6	266	1.7	1.7	
125.0	52	7.6	1.4	
120.1	57	2.9	0.6	G6
114.7	389	14.2	20.6	G2/5
107.7	623	0.9	2.1	A1
104.8	2103	2.6	18.4	
101.8	275	1.6	1.6	
100.4	907	1.4	4.6	M1
98.8	492	1.5	2.8	
96.5	477	0.7	1.2	β-Glc1
92.6	308	0.7	0.7	α-Glc1

Poplar

Chemical Shift [ppm]	Ampli-tude	Width [ppm]	Integral [%]	Carbon
181.0	109	6.7	3.1	
173.6	1037	3.2	14.1	Ac^{CO}
171.7	173	1.1	0.8	
152.6	744	3.8	12.2	S3/5
148.2	217	3.3	3.0	G3
145.2	151	2.3	1.5	G4
136.9	165	2.9	2.0	$S^{b}4$
134.2	315	3.8	5.1	$S^{a}4$
131.2	193	3.9	3.2	
120.0	42	2.4	0.4	
115.5	188	8.4	6.8	G5
108.9	16	1.9	0.1	
104.7	3650	2.6	40.1	$i/s/Xn1^{2f}$
102.4	60	0.5	0.1	$Xn1^{3f}$
100.6	632	2.7	7.2	
96.5	62	0.3	0.1	

Supplementary Table 6. Intermolecular interactions of polymers in intact plant stems. In the summary of intermolecular cross-peaks between different polymers of the secondary cell wall of woods, a total of 272 cross-peaks have been identified and categorized as 112 strong, 75 medium, and 85 weak interactions.

	Interactions	**s**	**m**	**w**	**Total**
Eucalyptus	cellulose-xylan	0	0	4	**4**
	cellulose-lignin	8	4	8	**20**
	lignin-lignin	4	11	4	**19**
	xylan-lignin	22	13	7	**42**
	mixed sugar-lignin	11	1	1	**13**
Poplar	cellulose-xylan	0	3	1	**4**
	cellulose-lignin	4	5	6	**15**
	lignin-lignin	2	6	8	**16**
	xylan-lignin	14	17	10	**41**
	mixed sugar-lignin	2	2	0	**4**
Spruce	cellulose-xylan	4	2	2	**8**
	cellulose-lignin	18	1	3	**22**
	lignin-lignin	0	0	0	**0**
	xylan-lignin	25	5	23	**53**
	Mannan-lignin	0	1	1	**2**
	Mannan-xylan	1	0	0	**1**
	mixed sugar-lignin	3	0	5	**8**
**Total**		**112**	**75**	**85**	**272**

Supplementary Table 7. Water-edited intensities of polysaccharide and lignin of Eucalyptus. The intensity ratio is obtained by comparing the water-edited and control 2D ^{13}C-^{13}C correlation spectra. Standard deviations of NMR signal-to-noise ratios are used as error bars. The numbers (bold) in parentheses indicate the average intensities under each type of the polysaccharide or lignin.

Type	cross peaks	Intensity
interior cellulose **(0.24)**	i4-1	0.44±0.03
	i4-3[a]	042±0.05
	i4-3[b,c]	0.42±0.03
	i4-2/5	0.42±0.09
	i4-6	0.40±0.07
	i6-1	0.48±0.19
	i6-4	0.4±0.2
	i6-3[a,b]	0.5±0.1
	i6-3[c]	0.5±0.1
	i6-2/5,2	0.5±0.1
surface cellulose **(0.36)**	s4-1	0.55±0.09
	s4-3/5	0.6±0.1
	s4-2	0.6±0.1
	s4-6	0.5±0.1
$xylan^{2f}$ **(0.31)**	Xn^{2f}4-1	0.52±0.09
	Xn^{2f}4-3	0.55±0.08
	Xn^{2f}4-5	0.47±0.09
	Xn^{2f}4-2	0.7±0.2
$xylan^{3f}$ **(0.44)**	Xn^{3f}2/3-1	0.7±0.2
	Xn^{3f}2/3-4	0.6±0.2
	Xn^{3f}2/3-5	0.6±0.2
	Xn^{3f}5-1	0.5±0.1
	Xn^{3f}5-4	0.5±0.1
lignin **(0.51)**	S3/5-S1[b]	0.50±0.08
	S3/5-S1[a]	0.48±0.03
	S3/5-S2/6[a]	0.61±0.04
	S3/5-S2/6[b]	0.49±0.09
	S3/5-OMe	0.53±0.08
	S'3/5-4	0.61±0.09
	S'3/5-2, G4-2	0.5±0.1
	S'3/5-OMe, G4-OMe	0.5±0.1
	S4-S3/5	0.46±0.09
	S4-S2/6	0.44±0.08
	S4-S1	0.47±0.08
	S4-OMe	0.51±0.09
	S2/6-S3/5	0.6±0.2
	S2/6-S1	0.5±0.2
	S2/6-S4	0.5±0.1
	S2/6-OMe	0.51±0.09
lignin OMe **(0.42)**	OMe-S3/5	0.42±0.06
	OMe-G3	0.43±0.07
	OMe-S1	0.40±0.09
	OMe-S4	0.5±0.1
	OMe-S2/6	0.4±0.1

Supplementary Table 8. Water-edited intensities of polymers in poplar. The intensity ratio is obtained by comparing the water-edited and control 2D ^{13}C-^{13}C correlation spectra. Standard deviations of NMR signal-to-noise ratios are used as error bars. The numbers (bold) in parentheses indicate the average intensities under each type of the polysaccharide or lignin.

Type	cross peaks	Absolute Intensity
interior cellulose **(0.11)**	i4-1	0.11±0.04
	i4-3[a]	0.09±0.07
	i4-3[b,c]	0.09±0.07
	i4-2/5	0.10±0.08
	i6-1	0.12±0.05
	i6-4	0.12±0.03
	i6-3[a,b]	0.11±0.03
	i6-3[c]	0.11±0.04
	i6-2/5	0.11±0.02
surface cellulose **(0.11)**	s4-1	0.12±0.06
	s4-3/5	0.10±0.04
	s4-2	0.11±0.04
	s4-6	0.11±0.03
$xylan^{2f}$ **(0.30)**	$Xn4^{2f}$-1	0.36±0.06
	$Xn4^{2f}$-3	0.42±0.06
	$Xn4^{2f}$-2	0.20±0.05
	$Xn4^{2f}$-5	0.21±0.06
$xylan^{3f}$ **(0.25)**	Xn^{3f}2/3-1	0.23±0.05
	Xn^{3f}2/3-4	0.39±0.09
	Xn^{3f}2/3-5	0.15±0.03
	Xn^{3f}5-1	0.31±0.07
	Xn^{3f}5-4	0.18±0.09
guaiacyl **(0.19)**	G5-3	0.14±0.04
	G5-1	0.14±0.03
	G5-OMe	0.18±0.02
	G2-3	0.28±0.06
	G2-1	0.16±0.05
	G2-6	0.21±0.04
	G2-OMe	0.21±0.03
syringyl **(0.16)**	S3/5-1[b]	0.16±0.09
	S3/5-1[a]	0.21±0.06
	S3/5-2/6	0.15±0.04
	S3/5-OMe	0.14±0.05
	S1[b]-S3/5	0.14±0.07
	S1[b]-S2/6	0.20±0.06
	S1[b]-OMe	0.15±0.06
	S1[a]-S3/5	0.13±0.03
	S1[a]-OMe	0.11±0.03
	S2/6-S3/5	0.17±0.04
	S2/6-S1[b]	0.20±0.06
	S2/6-OMe	0.11±0.03
syringyl/guaicyl OMe **(0.14)**	OMe-S3/5	0.13±0.04
	OMe-G3	0.14±0.03
	OMe-S1	0.14±0.04
	OMe-G5	0.15±0.05
	OMe-S2/6	0.15±0.03

Supplementary Table 9. Water-edited intensities of polysaccharides and lignin of spruce. The intensity ratio is obtained by comparing the water-edited and control 2D ^{13}C-^{13}C correlation spectra. Standard deviations of NMR signal-to-noise ratios are used as error bars. The numbers (bold) in parentheses indicate the average intensities under each type of the polysaccharide or lignin.

Type	cross peaks	Absolute Intensity
interior cellulose **(0.38)**	i4-1	0.44±0.02
	i4-3[a]	0.37±0.04
	i4-3[b,c]	0.37±0.03
	i4-2/5	0.39±0.03
	i6-1	0.42±0.07
	i6-4	0.4±0.1
	i6-3	0.32±0.05
	i6-2/5,2	0.4±0.1
surface cellulose **(0.43)**	s4-1	0.5±0.1
	s4-2/5	0.44±0.03
	s4-3	0.4±0.2
	s4-6	0.46±0.03
xylan^{2f} **(0.57)**	Xn^{2f}4-1	0.7±0.1
	Xn^{2f}4-3	0.43±0.04
	Xn^{2f}4-5	0.57±0.04
	Xn^{2f}4-1	0.67±0.05
	Xn^{2f}4-2	0.56±0.06
xylan ^{3f} **(0.40)**	Xn^{3f}2/3-1	0.4±0.1
	Xn^{3f}2/3-4	0.32±0.07
	Xn^{3f}2/3-5	0.4±0.1
	Xn^{3f}5-4	0.44±0.05
	Xn^{3f}5-1	0.48±0.04
mannan **(0.76)**	Man1-4	0.86±0.06
	Man1-5	0.63±0.06
	Man1-6	0.79±0.05
guaiacyl **(0.47)**	G3-1	0.4±0.1
	G3-2	0.47±0.04
	G3-OMe	0.52±0.09
	G1-3	0.5±0.1
	G6-3	0.40±0.06
	G6-OMe	0.35±0.03
	G2-3[b,c]	0.51±0.06
	G2-3[a]	0.65±0.05
	G2-OMe	0.54±0.15
guaiacyl OMe **(0.42)**	OMe-G3	0.45±0.08
	OMe-G1	0.40±0.06
	OMe-G2	0.42±0.07

Supplementary Table 10. ^{13}C-T_1 relaxation times of lignin and polysaccharides in the three woods. The data are fit using single exponential equations $I\ (t) = 1 - 2e^{-t/T}$ for T_1, DP (inversion recovery) and $I\ (t) = e^{-t/T}$ for T_1, CP (Torchia CP). Standard deviations of the fitting parameters are used as error bars. Unidentified (-).

	Eucalyptus				Poplar				Spruce			
	Atom (ppm)	T_1, CP (s)	Atom (ppm)	T_1, DP (s)	Atom (ppm)	T_1, CP (s)	Atom (ppm)	T_1, DP (s)	Atom (ppm)	T_1, CP (s)	Atom (ppm)	T_1, DP (s)
Ac^{CO}	174	2.1±0.1	174	1.6±0.2	174	2.83±0.05	174	2.4±0.2	174	1.4±0.2	174	1.3±0.1
S3/5	-	-	153	3.1±0.5	-	-	153	1.3±0.1	-	-	-	-
G3	148	4.6±0.2	-	-	148	4.2±0.2	-	-	148	3.7±0.1	148	-
G4/ S'3/5	145	4.7±0.3	145	1.8±0.2	145	4.1±0.1	145	2.4±0.2	145	3.6±0.1	145	2.4±0.2
S1	136	4.5±0.2	136	2.2±0.3	-	-	136	2.7±0.2	-	-	-	-
S4	134.5	4.7±0.3	134.5	1.9±0.3	134.5	4.2±0.1	134.5	2.7±0.2	-		-	
G1	-	-	-	-	-	-	-	-	130.8	2.9±0.1	130.8	0.9±0.1
G6	-	-	-	-	-	-	-	-	120.5	3.3±0.1	120.5	2.6±0.3
OMe	57	4.6±0.3	57	2.5±0.2	57	4.4±0.3	56.5	2.8±0.2	57	3.4±0.3	57	2.1±0.2
i/s/Xn^{2f}1	105	4.1±0.1	105	2.3±0.7	105	4.1±0.1	105	3.7±0.2	105	3.45±0.04	105	3.2±0.2
i4	89	4.8±0.2	89	4.4±0.2	89	4.8±0.2	89	4.3±0.2	89	3.97±0.06	89	3.9±0.2
s4	84	3.9±0.1	84	2.3±0.3	84	3.7±0.2	84	3.1±0.2	84	3.12±0.09	84	1.5±0.2
i6	65	3.7±0.3	65	2.9±0.3	65	3.6±0.3	65	3.4±0.3	65	3.0±0.2	65	2.4±0.3
s6	62	2.7±0.3	61	-	62	2.7±0.3	-	-	62	2.0±0.2	-	-
Xn^{3f}1	102.5	3.4±0.2	102.5	2.1±0.3	102.5	3.4±0.2	102.5	2.2±0.2	102.5	2.7±0.1	102.5	1.7±0.2
Man	-	-	-	-	-	-	-	-	100.2	2.3±0.1	100	0.8±0.1
Xn^{2f}4	82	3.7±0.2	82	1.4±0.2	82	3.7±0.2	82	2.1±0.4	81.8	2.8±0.1	82	1.1±0.2
Xn^{3f}4	78	3.6±0.2	78	1.2±0.2	78	3.6±0.2	78	1.4±0.3	78	2.5±0.1	78	0.8±0.1
$Xn^{2f/3f}Ac^{Me}$	21.5	3.9±0.1	21.5	2.2±0.2	21.5	4.7±0.2	21.5	1.5±0.1	21.5	2.1±0.2	21.5	1.1±0.1

Supplementary Table 11. ^{1}H-T$_{1\rho}$ relaxation times of lignin and polysaccharides in three samples. The data are fit using a single exponential equation: $I\ (t) = e^{-t/T}$. Standard deviations of the fitting parameters are used as error bars. Unidentified (-).

	Eucalyptus		Spruce		Poplar	
	Atom (ppm)	T$_1$ (ms)	Atom (ppm)	T$_1$ (ms)	Atom (ppm)	T$_1$ (ms)
S3/5	153.9	16.8±0.8	-	-	153.2	19.5±0.6
G1^{a}	-	-	131	8.3±0.6	-	-
G1$^{b/c}$	-	-	134	11.6±0.5	-	-
G1^{d}	-	-	136	10.1±0.7	-	-
G3^{a}	148.1	10±1	152.2	13±1	148.1	14±1
G3^{c}	-	-	147.6	12.8±0.9	-	-
S'3/5/G4	145.6	11.4±0.8	-	-	145.0	13.8±0.9
S4	135.9	12±1	-	8.0±0.5	-	-
G1	-	-	134.2	8.9±0.7	-	-
S'^{b}4	130.1	11.1±0.7	-	-	-	-
G6	-	-	119.0	8.8±0.6	-	-
G5	115.3	8.0±0.7	116.3	8.2±0.7	116.2	9.1±0.8
G2	-	-	113.5	8.3±0.6	-	-
S'1	110.9	10.1±0.9	-	-	-	-
OMe	57	14.2±0.7	57	12.5±0.7	57	15.1±0.8
i/s/Xn1^{2f}	105.0	28.1±0.9	105.0	29±2	105.0	36±2
i4	89.0	35±1	89.0	35±1	89.0	43±2
s4	84.2	22 ±1	84.1	20±1	84.5	28±2
i6	65.0	26±2	65.0	26±2	64.8	32±2
s6	61.5	16±1	61.5	15±1	61.5	19±1
Xn1^{3f}	102.5	12.8±0.9	102.3	12±1	102.4	13.0±0.8
Man1	-	-	101	10.4±0.9	-	-
Xn4^{2f}	82.0	14.6±0.9	82.0	13.7±0.9	81.8	17±1
Xn4^{3f}	78.1	11.5±0.9	78.0	10.2±0.9	78.0	12±1
Xn^{2f,3f}Ac^{Me}	21.0	17.6±0.6	21.0	13±1	21.5	19.5±0.5

Supplementary Table 12. Parameters of 1D solid-state NMR experiments measured on the three wood samples. Recycle delay (d1); number of scans (NS); number of points of time domain for the direct (td2) and indirect (td1) dimensions; the acquisition time of the direct dimension (aq2); the evolution time of indirect dimension (aq1); excitation frequency for proton (ν^1H) and carbon ($\nu^{13}C$) channels.

	CP	DP	Quantitative DP	^{13}C-T_1 Torchia	^{13}C-T_1 Inversion-recovery	^1H-$T_{1\rho}$	1D Water-edited
Field (T)	14.1	14.1	14.1	9.4	9.4	9.4	9.4
Temp. (K)	294	294	294	300	300	300	278
MAS (kHz)	14	14	14	10	10	10	10
d1(s)	1.7	2	35/40[a]	2	30	2	1.8
NS	128	128	64	256	128	256/128/64[b]	512
td2	2048	2800	2800	1600	1600	1400	1400
aq2 (ms)	14	19.6	19.6	16	16	14	14
ν^1H (kHz)	83.3	-	-	62.5	-	62.5	83.3
$\nu^{13}C$ (kHz)	62.5	62.5	62.5	62.5	62.5	-	62.5
CP ($^1H/^{13}C$) (kHz)	62.5	-	-	62.5	-	51[c]	62.5
CP contact time (ms)	1	-	-	1	-	1.0	1.0
^1H decoupling (kHz)	83.3	83.3	83.3	62.5	83.3	62.5	83.3
Processing	GM (-7, 0.07)	GM (-7, 0.07)	GM (-7, 0.07)	GM (-7, 0.07)	GM (-7, 0.07)	GM (-5, 0.1)	GM (-5, 0.1)

[a]Recycle delays are 40s (for eucalyptus) and 35s (for the other two). [b]To protect the probe during longer LG-SL time (14 ms and 19 ms), smaller NSs are used (128/64). [c]For ^1H-$T_{1\rho}$, 51 kHz for CP matching means $w1_{eff}$ is 62.5 kHz during LG-SL and LG-CP.

Supplementary Table 13. Parameters of 2D NMR experiments measured on the three wood samples. Recycle delay (d1); number of scans (NS); number of points of time domain for the direct (td2) and indirect (td1) dimensions; the acquisition time of the direct dimension (aq2); the evolution time of indirect dimension (aq1); excitation frequency for proton (ν^1H) and carbon ($\nu^{13}C$) channels.

	CP-INADQ	DP-INADQ	RFDR	Dipolar-gated PDSD	2D Water-edited	DARR	DIPSHIFT	DP-PDSD	HSQC
Field (T)	14.1	14.1	9.4	14.1	9.4	9.4	9.4	9.4	11.8
Temp. (K)	294	294	298	294	278	278	298	298	-
MAS (kHz)	14	14	10	14	10	10	7.5	12	-
d1(s)	1.6	1.6	2.0	1.48	1.65	1.65	2.0	1.7	1
NS	32	16	128	160	128/256 [a]	128	128	192	224
td2	1400	1800	1600	1400	1400	1400	1600	1600	2048
td1	340	112	280	180	152	152	13	200	256
aq2 (ms)	14	18	16	14	14	14	16	16	12.78
aq1 (ms)	4.5	4.5	7.00	3.42	4.94	4.94	0.14	4.20	3.91
ν^1H (kHz)	83.3	-	71.4	83.3	83.3	83.3	83.3	-	31.3
$\nu^{13}C$ (kHz)	62.5	62.5	62.5	62.5	62.5	62.5	62.5	62.5	20.8
CP ($^1H/^{13}C$) (kHz)	62.5	-	62.5	62.5	62.5	62.5	62.5	-	-
CP contact time (ms)	1	-	1	1	1	1	1	-	-
^1H decoupling (kHz)	83.3	83.3	71.4	83.3	83.3	83.3	83.3	83.3	31.3
Mixing time (ms)	-	-	1.6	20/100/1000 [b]	50	50	-	100	0.86[c]
Processing	QSINE (ssb:2.8)	QSINE (ssb:3)	GM (-10, 0.05) QSINE (ssb:4)[e]	GM (-15, 0.04) QSINE (ssb:3)[e]	GM (-15, 0.04) QSINE (ssb:4.5)[e]	GM (-30, 0.03) QSINE (ssb:4.5)[e]	GM (-5, 0.1)	GM (-30, 0.03) QSINE (ssb:4)[e]	QSINC (ssb:3.5) QSINE (ssb:3.5)[d]

[a] 128 NS for control one and 256 NS for 4ms-SD water edited one. [b] 1000ms/100ms for long and short mixing time and spruce also has 20-ms mixing time spectrum. [c] $1/(8J_{CH})$. [d] Along direct and indirect dimensions respectively. For the later forward linear prediction is applied with 32 coefficients. [e] GM for lignin region and QSINE for carbohydrate region.

Supplementary Table 14. Parameters of ssNMR experiments measured for never-dried spruce and eucalyptus samples. The same experiments with same parameters were measured again for both two wood samples after lyophilization and rehydration. Never-dried spruce was involved in all 1D and 2D experiments, and never-dried eucalyptus was only involved in 1D experiments. Recycle delay (d1); number of scans (NS); number of points of time domain for the direct (td2) and indirect (td1) dimensions; the acquisition time of the direct dimension (aq2); the evolution time of indirect dimension (aq1); excitation frequency for proton (ν^1H) and carbon (ν^{13}C) channels.

	CP	DP	Quantitative DP	^{13}C-T_1 Torchia	^{1}H-$T_{1\rho}$	1D Water-edited	2D Water-edited	CP-INADQ	DP-INADQ	53-ms CORD
Field (T)	9.4	9.4	9.4	9.4	9.4	9.4	9.4	9.4	9.4	9.4
Temp. (K)	298	298	298	298	298	280	280	298	298	298
MAS (kHz)	10	10	10	10	10	10	10	10	10	10
d1(s)	2	2	35	2	2	2	1.7	1.8	2	2
NS	1k	1k	512	512	512/256/128[a]	1k/512	96	192	192	128
td2	1600	1600	1600	1600	1600	1400	1600	1400	1600	1600
td1	1	1	1	1	1	1	166	90	90	166
aq2 (ms)	16	16	16	16	16	14	16	14	16	16
aq1 (ms)	-	-	-	-	-	-	4.15	5.31	5.31	4.15
ν^1H (kHz)	83.3	-	-	83.3	62.5	83.3	83.3	83.3	-	83.3
ν^{13}C (kHz)	62.5	62.5	62.5	62.5	-	62.5	62.5	62.5	62.5	62.5
CP match (^{1}H/^{13}C) (kHz)	62.5	-	-	62.5	51[b]	62.5	62.5	62.5	-	62.5
CP contact time (ms)	1.0	-	-	1.0	1.0	1.0	1.0	1.0	-	1.0
^{1}H decoupling (kHz)	83.3	83.3	83.3	83.3	62.5	83.3	83.3	83.3	83.3	83.3
Processing	GM (-5, 0.1)	GM (-10, 0.05)	GM (-10, 0.05)	GM (-5, 0.1)	GM (-5, 0.1)	GM (-5, 0.1)	GM (-20, 0.04) QSINE (ssb:4)[c]	QSINE (ssb:4)	QSINE (ssb:4)	GM (-30, 0.03)

[a] To protect the probe during longer LG-SL time (14 ms and 19 ms), smaller NSs are used (256/128). [b] For ^{1}H-$T_{1\rho}$, 51 kHz for CP matching means $w1_{eff}$ is 62.5 kHz during LG-SL and LG-CP. [c] GM for lignin region and QSINE for carbohydrate region.

Supplementary Reference

1. Purushotham, P., Ho, R. & Zimmer, J. Architecture of a catalytically active homotrimeric plant cellulose synthase complex. *Science* **369**, 1089-1094 (2020).
2. Yang, H. & Kubicki, J. A density functional theory study on the shape of the primary cellulose microfibril in plants: effects of C6 exocyclic group conformation and H-bonding. *Cellulose* **27**, 2389-2402 (2020).
3. Phyo, P., Wang, T., Yang, Y., O'Neill, H. & Hong, M. Direct Determination of Hydroxymethyl Conformations of Plant Cell Wall Cellulose Using ^{1}H Polarization Transfer Solid-State NMR. *Biomacromolecules* **19**, 1485-1497 (2018).
4. Wang, T., Yang, H., Kubicki, J.D. & Hong, M. Cellulose Structural Polymorphism in Plant Primary Cell Walls Investigated by High-Field 2D Solid-State NMR Spectroscopy and Density Functional Theory Calculations. *Biomacromolecules* **17**, 2210-2222 (2016).
5. Massiot, D. et al. Modelling one- and two-dimensional solid-state NMR spectra. *Magn Reson Chem* **40**, 70-76 (2002).
6. Yang, H. et al. Quantum Calculations on Plant Cell Wall Component Interactions. *Interdis. Sci.* **11**, 485-495 (2019).
7. Rencoret, J. et al. HSQC-NMR analysis of lignin in woody (Eucalyptus globulus and Picea abies) and non-woody (Agave sisalana) ball-milled plant materials at the gel state 10th EWLP, Stockholm, Sweden, August 25–28, 2008. *Holzforschung* **63**, 691-698 (2009).
8. Rico, A., Rencoret, J., del Rio, J.C., Martinez, A.T. & Gutierrez, A. In-Depth 2D NMR Study of Lignin Modification During Pretreatment of Eucalyptus Wood with Laccase and Mediators. *Bioenergy Res.* **8**, 211-230 (2015).
9. Ibarra, D. et al. Structural modification of eucalypt pulp lignin in a totally chlorine-free bleaching sequence including a laccase-mediator stage. *Holzforschung* **61**, 634-646 (2007).
10. Rencoret, J. et al. Lignin Composition and Structure in Young versus Adult Eucalyptus globulus Plants. *Plant Physiol.* **155**, 667-682 (2011).
11. Wen, J.L., Sun, S.L., Yuan, T.Q., Xu, F. & Sun, R.C. Structural elucidation of lignin polymers of Eucalyptus chips during organosolv pretreatment and extended delignification. *J. Agric. Food Chem.* **61**, 11067-11075 (2013).
12. Yuan, T.-Q., Sun, S.-N., Xu, F. & Sun, R.-C. Characterization of Lignin Structures and Lignin–Carbohydrate Complex (LCC) Linkages by Quantitative ^{13}C and 2D HSQC NMR Spectroscopy. *J. Agric. Food Chem.* **59**, 10604-10614 (2011).
13. Li, M. et al. The effect of liquid hot water pretreatment on the chemical–structural alteration and the reduced recalcitrance in poplar. *Biotechnol. Biofuels* **10**, 237 (2017).
14. Samuel, R. et al. HSQC (heteronuclear single quantum coherence) 13C–1H correlation spectra of whole biomass in perdeuterated pyridinium chloride–DMSO system: An effective tool for evaluating pretreatment. *Fuel* **90**, 2836-2842 (2011).
15. Samuel, R. et al. Investigation of the fate of poplar lignin during autohydrolysis pretreatment to understand the biomass recalcitrance. *RSC Adv.* **3**, 5305-5309 (2013).
16. Wang, H.M. et al. Structural Variations of Lignin Macromolecules from Early Growth Stages of Poplar Cell Walls. *ACS Sustainable Chem. Eng.* **8**, 1813-1822 (2020).
17. Lagerquist, L. et al. Structural and thermal analysis of softwood lignins from a pressurized hot water extraction biorefinery process and modified derivatives. *Molecules* **24**, 335 (2019).
18. Michalak, L., Knutsen, S.H., Aarum, I. & Westereng, B. Effects of pH on steam explosion extraction of acetylated galactoglucomannan from Norway spruce. *Biotechnol. Biofuels* **11**, 1-12 (2018).
19. Du, X. et al. Analysis of lignin-carbohydrate and lignin-lignin linkages after hydrolase treatment of xylan-lignin, glucomannan-lignin and glucan-lignin complexes from spruce wood. *Planta* **239**, 1079-1090 (2014).
