# Supplementary material for: Carbohydrate-aromatic interface and molecular architecture of lignocellulose
Source: Nat Commun. 2022 Jan 27;13:538. doi: 10.1038/s41467-022-28165-3 (PMC8795156; doi:10.1038/s41467-022-28165-3)
Supplement: Supplementary file 3 — Description of Additional Supplementary Files [file 41467_2022_28165_MOESM3_ESM.docx]

**Description of Additional Supplementary files**

**Supplementary Data 1**.

Description: ^13^C chemical shifts of rigid polysaccharides and lignin in wood secondary cell walls. The rigid components are identified from ^13^C CP-based INADEQUATE and RFDR spectra. Superscripts are used to denote different allomorphs. Unidentified signals are indicated as (-). Not applicable (/)

**Supplementary Data 2.**

Description: The intensities of intermolecular cross peaks of eucalyptus*.* In sum, 98 intermolecular interactions are identified, including 45 strong, 29 medium, and 24 weak restraints. In gated 1-s PDSD spectra, a peak higher than 4% is categorized as a strong restraint (s, in bold), between 2% - 4% for medium (m) restraint, and below 2% for weak (w) restraint. All the peaks shown in the gated 100-ms PDSD spectrum are categorized as strong restraints. The intensity is a relative ratio of the peak, which is normalized by the integral of the ^13^C cross-section.

**Supplementary Data 3.**

Description: The intensities of intermolecular cross peaks of poplar wood*.* In sum, 80 intermolecular interactions are identified, including 22 strong, 33 medium, and 25 weak restraints. In gated 1-s PDSD spectra, a peak higher than 4% is categorized as a strong restraint (s, in bold), between 2% - 4% for medium (m) restraint, and below 2% for weak (w) restraint. All the peaks shown in the gated 100-ms PDSD spectrum are categorized as strong restraints. The intensity is a relative ratio of the peak, which is normalized by the integral of the ^13^C cross-section.

**Supplementary Data 4.**

Description: The intensities of intermolecular cross peaks of spruce*.* In sum, 94 intermolecular interactions are identified, including 51 strong, 9 medium, and 34 weak restraints. In gated 1-s PDSD spectra, a peak higher than 4% is categorized as a strong restraint (s, in bold), between 2% - 4% for medium (m) restraint, and below 2% for weak (w) restraint. All the peaks shown in the gated 100-ms PDSD spectrum are categorized as strong restraints. The intensity is a relative ratio of the peak, which is normalized by the integral of the ^13^C cross-section.

**Supplementary Data 5.**

Description: Comparison of ^13^C chemical shifts with literature-reported values. The comparison results are shown for carbohydrates, lignin aromatic carbons as well as covalent linkers. The references are indicated for each comparison.
