# Supplementary material for: Carbohydrate-aromatic interface and molecular architecture of lignocellulose
Source: Nat Commun. 2022 Jan 27;13:538. doi: 10.1038/s41467-022-28165-3 (PMC8795156; doi:10.1038/s41467-022-28165-3)
Supplement: Supplementary file 9 — Reporting Summary [file 41467_2022_28165_MOESM9_ESM.pdf]

nature portfolio

Corresponding author(s): Tuo Wang

Last updated by author(s): Dec 17, 2021

Reporting Summary

Nature Portfolio wishes to improve the reproducibility of the work that we publish. This form provides structure for consistency and transparency in reporting. For further information on Nature Portfolio policies, see our Editorial Policies and the Editorial Policy Checklist.

Statistics

For all statistical analyses, confirm that the following items are present in the figure legend, table legend, main text, or Methods section.

n/a	Confirmed	
☐	☒	The exact sample size (*n*) for each experimental group/condition, given as a discrete number and unit of measurement
☐	☒	A statement on whether measurements were taken from distinct samples or whether the same sample was measured repeatedly
☒	☐	The statistical test(s) used AND whether they are one- or two-sided<br>*Only common tests should be described solely by name; describe more complex techniques in the Methods section.*
☒	☐	A description of all covariates tested
☐	☒	A description of any assumptions or corrections, such as tests of normality and adjustment for multiple comparisons
☐	☒	A full description of the statistical parameters including central tendency (e.g. means) or other basic estimates (e.g. regression coefficient) AND variation (e.g. standard deviation) or associated estimates of uncertainty (e.g. confidence intervals)
☒	☐	For null hypothesis testing, the test statistic (e.g. *F*, *t*, *r*) with confidence intervals, effect sizes, degrees of freedom and *P* value noted<br>*Give P values as exact values whenever suitable.*
☒	☐	For Bayesian analysis, information on the choice of priors and Markov chain Monte Carlo settings
☒	☐	For hierarchical and complex designs, identification of the appropriate level for tests and full reporting of outcomes
☒	☐	Estimates of effect sizes (e.g. Cohen's *d*, Pearson's *r*), indicating how they were calculated

Our web collection on statistics for biologists contains articles on many of the points above.

Software and code

Policy information about availability of computer code

Data collection	The data include NMR spectra collected using Bruker Topspin software (Versions 3.5 and 2.1).
Data analysis	NMR data were analyzed using Bruker Topspin software 4.0.8 and Dmfit software (Version dmfit#20211004). Relaxation and hydration data are fit using Microsoft Excel 365 and OriginPro 8. Data are plotted using Adobe illustrator CC 18.0. Gaussian 09 was used in DFT calculation.

For manuscripts utilizing custom algorithms or software that are central to the research but not yet described in published literature, software must be made available to editors and reviewers. We strongly encourage code deposition in a community repository (e.g. GitHub). See the Nature Portfolio guidelines for submitting code & software for further information.

Data

Policy information about availability of data

All manuscripts must include a data availability statement. This statement should provide the following information, where applicable:

- Accession codes, unique identifiers, or web links for publicly available datasets
- A description of any restrictions on data availability
- For clinical datasets or third party data, please ensure that the statement adheres to our policy

All NMR spectra and biochemical data that support the findings of this study are provided in the article, supplementary information, and Source Data files. The processed topspin NMR datasets of plant stems are available upon requests from the corresponding author due to the large size of the files and lack of an appropriate repository. The Source Data underlying Figs. 1, 2, 3, 4, and 5 as well as Supplementary Figs 8, 9, 12, 14, 15, and 16 are provided as a Source Data file.

Field-specific reporting

Please select the one below that is the best fit for your research. If you are not sure, read the appropriate sections before making your selection.

☒ Life sciences ☐ Behavioural & social sciences ☐ Ecological, evolutionary & environmental sciences

For a reference copy of the document with all sections, see nature.com/documents/nr-reporting-summary-flat.pdf

Life sciences study design

All studies must disclose on these points even when the disclosure is negative.

Sample size	Each NMR sample contains 30-100 mg (hydrated mass) of plant materials, which is determined by weighing the material before and after packing into a NMR rotor. The NMR spectra report the average feature of all cells in each sample. A large number of scans were collected for averaging and for each NMR spectrum, resulting in reproducibility of each spectrum. The exact number of scans are summarized in Supplementary Tables 12-14.<br>In total, seven different plant samples were measured, including the rehydrated samples of poplar, eucalyptus, and spruce, the never-dried samples of spruce and eucalyptus, as well as the dehydrated-and-then-rehydrated samples of spruce and eucalyptus.
Data exclusions	There were no data exclusions.
Replication	Each NMR spectra were averaged with a large number of scans (Supplementary Tables 16-18) , being intrinsically reproducible. All attempts for replication were successful.
Randomization	Different plant species were studied separately.
Blinding	Not Applicable to NMR studies of the fundamental structure of carbohydrate molecules and lignin. As the samples studied are natural mixtures of molecules, there is no differentiation and there is no need for randomized controlled trial.

Reporting for specific materials, systems and methods

We require information from authors about some types of materials, experimental systems and methods used in many studies. Here, indicate whether each material, system or method listed is relevant to your study. If you are not sure if a list item applies to your research, read the appropriate section before selecting a response.

Materials & experimental systems

n/a	Involved in the study	
☒	☐	Antibodies
☒	☐	Eukaryotic cell lines
☒	☐	Palaeontology and archaeology
☒	☐	Animals and other organisms
☒	☐	Human research participants
☒	☐	Clinical data
☒	☐	Dual use research of concern

Methods

n/a	Involved in the study	
☒	☐	ChIP-seq
☒	☐	Flow cytometry
☒	☐	MRI-based neuroimaging
